# Influences of different referral modes on clinical outcomes after endovascular therapy for acute ischemic stroke

**DOI:** 10.1186/s12883-022-02751-w

**Published:** 2022-06-21

**Authors:** Jie Hou, Zhi-liang Guo, Zhi-chao Huang, Huai-shun Wang, Shou-jiang You, Guo-dong Xiao

**Affiliations:** Department of Neurology and Clinical Research Center of Neurological Disease, The Second Affiliated Hospital of Soochow University, Suzhou, 215004 Jiangsu China

**Keywords:** Referral mode, Ischemic Stroke, Endovascular therapy, Outcomes

## Abstract

**Background and purpose:**

As endovascular thrombectomy (EVT) is time-dependent, it is crucial to refer patients promptly. Current referral modes include Mothership (MS), Drip and Ship (DS) and Drive the Doctor (DD). The purpose of this study was to investigate the influences of different referral modes on the clinical outcomes of patients with acute ischemic stroke after EVT.

**Methods:**

A total of 349 patients from 15 hospitals between April 2017 and March 2020 were enrolled. The primary outcomes include poor outcome (modified Rankin Scale score of 3 to 6), symptomatic intracranial hemorrhage transformation (sICH), mortality and cost. Regression analysis was used to assess the association of referral modes with poor outcome, sICH, mortality and cost in acute ischemic stroke patients.

**Results:**

Among the 349 patients, 83 were in DD group (23.78%), 85 in MS group (24.36%) and 181 in DS group (51.86%). There were statistically significant differences in intravenous thrombolysis, onset-to-door time, onset-to-puncture time, puncture-to-recanalization time, door-to-puncture time, door-to-recanalization time, and cost among the DD, MS, and DS groups (59.04% vs 35.29% vs 33.15%, *P*<0.001; 90 vs 166 vs 170 minutes, *P*<0.001; 230 vs 270 vs 270 minutes, *P*<0.001; 82 vs 54 vs 51 minutes, *P*<0.001; 110 vs 85 vs 96 minutes, *P*=0.004; 210 vs 146 vs 150 minutes, *P*<0.001; 64258 vs 80041 vs 70750 Chinese Yuan, *P*=0.018). In terms of sICH, mortality and poor outcome, there was no significant difference among the DD, MS, and DS groups (22.89% vs 18.82% vs 19.34%, *P*=0.758; 24.10% vs 24.71% vs 29.83%, *P*=0.521; 64.47% vs 64.71% vs 68.51%, *P*=0.827). The results of multiple regression analysis indicated that there was no independent correlation between different referral modes regarding sICH (*OR*_MS_: 0.50, 95%CI: 0.18, 1.38, *P*=0.1830; *OR*_DS_: 0.47, 95%CI: 0.19, 1.16, *P*=0.1000), mortality (*OR*_MS_: 0.56, 95%CI: 0.19, 1.67, *P*=0.2993; *OR*_DS_: 0.65, 95%CI: 0.25, 1.69, *P*=0.3744) and poor outcome (*OR*_MS_: 0.61, 95%CI: 0.25, 1.47, *P*=0.2705; *OR*_DS_: 0.53, 95%CI: 0.24, 1.18, *P*=0.1223). However, there was a correlation between MS group and cost (*β*=30449.73, 95%CI: 11022.18, 49877.29; *P=*0.0023). The multiple regression analysis on patients finally admitted in comprehensive stroke center (MS+DS) versus patients finally admitted in primary stroke center (DD) showed that DD mode was independently associated with lower costs (*β*=-19438.86, 95%CI: -35977.79, -2899.94; *P*=0.0219).

**Conclusion:**

There was no independent correlation between three referral modes and sICH, mortality, poor outcome correspondingly. Different referral modes can be implemented in clinical practice according to the situations encountered. Compared to MS and DS modes, DD mode is more economical.

**Supplementary Information:**

The online version contains supplementary material available at 10.1186/s12883-022-02751-w.

## Introduction

Stroke is one of the most common causes of death and the most frequent cause of disability in the world [1]. Time is the brain, and the treatment of stroke is highly time-dependent [2]. Moreover, a considerable number of patients are unable to reach the hospital in time [1, 3]. Endovascular therapy has been proved to be effective in ischemic stroke patients with large vessel occlusion (LVO), significantly superior to conventional stroke treatment [4–11]. Timely referral of patients is very important. The National Institute of Neurological Diseases and Stroke (NINDS) defines the time standard for the in-hospital emergency chain after stroke patients arrive at hospitals [12]. Thus NINDS's recommendation scope only covers the in-hospital delays. However, the majority delay of patients arrived at stroke center occurred in the pre-hospital phase, and the time delay in the process of transport is the main reason for patients to exceed the time window of endovascular therapy [13, 14]. Therefore, shortening the pre-hospital time delay in patients with acute ischemic stroke is crucial. As a result, three clinical referral modes are derived for ischemic stroke patients with LVO: Mothership mode (MS, patients were referred directly to the comprehensive stroke center), Drip and Ship mode (DS, patients were referred to a primary stroke center and then to a comprehensive stroke center), and Drive the Doctor mode (DD, the physicians in comprehensive stroke center were sent to the primary stroke center) [15–19].

There is still a lack of high-quality evidence to clarify the prognostic significance of the three referral modes for endovascular therapy until present, especially the DD mode. The main purpose of our study was to analyze the effects of three different referral modes on the outcomes (symptomatic intracranial hemorrhage transformation, mortality, poor outcome and cost) of patients with acute ischemic stroke after endovascular treatment.

## Method

### Research subjects

**Fig. 1:**
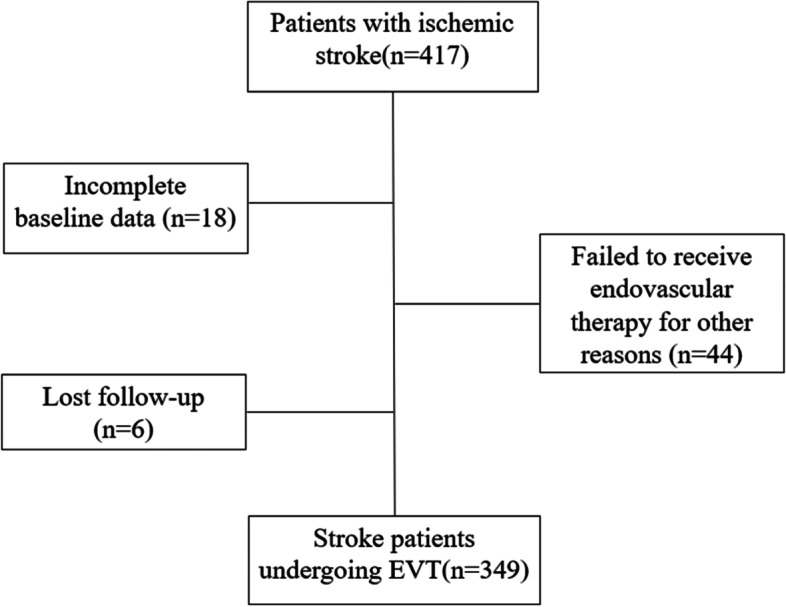
Flow chart of included and excluded patients

This was a retrospective study with multi-facility-cooperation. A total of 417 patients with acute ischemic stroke were enrolled from 15 hospitals between April 2017 and March 2020. We excluded 68 patients based on exclusion criteria (eighteen patients with incomplete baseline data, six patients lost follow-up, forty-four patients failed to receive endovascular therapy including one patient with spontaneous recanalization, one patient died preoperatively, five patients with failed approach and thirty-seven with absence of LVO or intra-arterial thrombolytic alone), and 349 patients met inclusion criteria eventually (Figure 1). According to the different referral modes, the patients were divided into three groups: DD mode, MS mode and DS mode. Patients with DS mode and MS mode were defined as group A (traditional referral modes), and patients with DD mode were defined as group B (emerging referral mode) based on patients’ final admission to the stroke center.

**Inclusion criteria:** (1) acute ischemic stroke patients with large vessels occlusion. (2) received endovascular therapy and signed informed consent.

**Exclusion criteria:** (1) patients with incomplete baseline data or lost follow-up. (2) patients who failed to receive endovascular therapy for other reasons. (3) patients with previous neurological or psychiatric diseases. (4) previous mRS score > 1.

### Operation procedures

The stroke centers in this study were dominated by the comprehensive stroke center (CSC) of our hospital and radiated outward to the other 14 primary stroke centers (PSC). The PSCs have adequate endovascular treatment equipment to ensure the operation, but the availability of neurointerventional surgeons were limited for them. Therefore, our hospital is equipped with 7 days/24-hour professional neurointerventional surgeons to meet the medical needs of acute ischemic stroke patients with different referral modes. All patients were treated with endovascular therapy by the doctors of the neurointervention team in our hospital. Forming a regional and unique first-aid circle for endovascular therapy of stroke.

### Data collection

#### General baseline and history data

Gender, age, NIHSS, ASPECTS, systolic blood pressure, diastolic blood pressure, pulse, infarction location, past medical history, TOAST classification and intravenous thrombolysis.

#### The operation-related Indicators

The location of occlusive vessels, the methods of treatment, onset-to-door time (ODT), onset-to-puncture time (OPT), onset-to-recanalization time (ORT), puncture-to-recanalization time (PRT), door-to-puncture time (DPT), door-to-recanalization time (DRT) [15, 16, 18], recanalization rate, intracranial hemorrhage transformation, cost and mRS.

#### Evaluation indicators

The outcomes of interest include symptomatic intracranial hemorrhage transformation (sICH), mortality, poor outcome and cost. Neurological deficits were assessed according to the National Institutes of Health Stroke Scale (NIHSS) [20]. Early intracranial ischemic changes were evaluated using Albert Stroke Project Early CT Score (ASPECTS) [21]. Vascular recanalization was defined as modified Thrombolysis in Cerebral Infarction Score (mTICI) 2B-3 [22]. Good outcome was defined as 3-month modified Rankin Scale (mRS)≤2. Poor outcome was defined as 3-month mRS≥3. Mortality was defined as death from any causes within 90 days [23]. sICH was defined as any type of intracranial hemorrhage with an increase in NIHSS of greater than or equal to 4 points or death within 24 hours [24, 25].

#### Statistical analysis

Using SPSS 23.0 and EmpowerStats software to input and analyze the data. Statistical differences between groups were determined using one-way ANOVA (measure variables of the normal distribution), Kruskal-Wallis H (measure variables of the skewness distribution) and Chi-square test (categorical variables). Multiple regression analysis of three modes was constructed. Model I adjusted for age and gender, and Model II adjusted for variables that were significantly correlated with clinical outcomes (*P*<0.1) or changed by more than 10% compared with the initial regression coefficient (variables to be adjusted according to covariate screening) [26]. Stratified analyses were performed using stratified regression models to assess the stability of associations between different referral modes and clinical outcomes. *P*≤0.05 was considered to be statistically significant.

## Results

### Clinical Baseline

#### Baseline characteristics

Table 1 showed that there were 83 cases (23.78%) in the DD group, 85 cases (24.36%) in the MS group and 181 cases (51.86%) in the DS group. The male patients were 52 (62.65%), 46 (54.12%) and 110 (60.77%), respectively, with an average age of 66.77 years (12.69),66.61 years (11.62) and 64.67 years (14.57). There were significant differences in ASPECTS, pulse, intravenous thrombolysis among the three groups (*P*<0.05). There were no significant differences in NIHSS, systolic blood pressure, diastolic blood pressure, risk factors, TOAST classification, location of vascular occlusion, location of infarction and methods of treatment among the three groups(*P*>0.05). The proportion of intravenous thrombolysis in the DD group was higher than that in the MS and DS groups (59.04% vs 35.29% vs 33.15 %, *P*<0.001) (Table 1).

**Table 1:** Baseline data among three groups of referral modes

**Mode**	**DD** ***N*=83**	**MS** ***N*=85**	**DS** ***N*=181**	***P*-value**
**Male(n,%)**	52 (62.65%)	46 (54.12%)	110 (60.77%)	0.476
**Age, X(SD)**	66.77 (12.69)	66.61 (11.62)	64.67 (14.57)	0.375
**NIHSS, M(IQR)**	15.00 (11.00-19.00)	16.00 (12.00-19.00)	16.00 (13.00-20.00)	0.253
**ASPECTS, M(IQR)**	7.00 (7.00-8.00)	7.00 (6.00-7.00)	7.00 (6.00-7.00)	<0.001
**Pulse, X (SD)**	78.04 (17.28)	84.01 (18.05)	82.65 (18.88)	0.027
**Systolic BP, X (SD)**	150.17 (21.28)	148.73 (27.23)	148.69 (21.80)	0.831
**Diastolic BP, X (SD)**	87.77 (15.09)	85.65 (17.88)	86.89 (15.90)	0.358
**Risk factor (n,%)**				
**Hypertension**	51 (61.45%)	56 (65.88%)	128 (70.72%)	0.312
**Diabetes mellitus**	20 (24.10%)	11 (12.94%)	36 (19.89%)	0.175
**Atrial fibrillation**	35 (42.17%)	41 (48.24%)	76 (41.99%)	0.606
**Coronary heart disease**	6 (7.23%)	6 (7.06%)	19 (10.50%)	0.545
**Hyperlipemia**	30 (36.14%)	20 (23.53%)	64 (35.36%)	0.118
** Gout**	4 (4.82%)	1 (1.18%)	8 (4.42%)	0.357
**Previous stroke**	20 (24.10%)	14 (16.47%)	35 (19.34%)	0.453
**Smoking**	22 (26.51%)	27 (31.76%)	51 (28.18%)	0.737
**Drinking**	16 (19.28%)	22 (25.88%)	39 (21.55%)	0.570
**TOAST(n,%)**				0.377
**Large-artery atherosclerosis**	41 (49.40%)	30 (35.29%)	85 (46.96%)	
**Cardioembolism**	37 (44.58%)	49 (57.65%)	86 (47.51%)	
**Other/undetermined etiology**	5 (6.02%)	6 (7.06%)	10 (5.52%)	
**Procedural characteristics**				
**Occlusion site(n,%)**				0.092
**Intracranial ICA**	27 (32.53%)	13 (15.29%)	53 (29.28%)	
**MCA**	46 (55.42%)	61 (71.76%)	105 (58.01%)	
**Vertebral/basilar artery**	10 (12.05%)	11 (12.94%)	23 (12.71%)	
**Infarction site(n,%)**				0.414
**Left**	50 (60.24%)	43 (50.59%)	96 (53.04%)	
**Right**	33 (39.76%)	42 (49.41%)	85 (46.96%)	
**IV(n,%)**	49 (59.04%)	30 (35.29%)	60 (33.15%)	<0.001
**Threapy(n,%)**				0.059
**Stent**	35 (42.17%)	42 (49.41%)	85 (46.96%)	
**Aspiration**	3 (3.61%)	11 (12.94%)	20 (11.05%)	
**Stent combined aspiration**	9 (10.84%)	10 (11.76%)	28 (15.47%)	
**Other**	36 (43.37%)	22 (25.88%)	48 (26.52%)	

*X* Mean, *SD* Standard deviation, *M* Median, *IQR* Interquartile range, *DD* Drive the Doctor, *MS* Mothership, *DS* Drip and Ship, *NIHSS* National Institutes of Health Stroke Scale, *ASPECTS* Albert Stroke Project Early CT Score, *BP* Blood pressure, *TOAST* Trial of Org 10172 in Acute Stroke Treatment, *ICA* Internal carotid artery, *MCA* Middle cerebral artery, *IV* Intravenous thrombolysis.

#### Outcomes after endovascular thrombectomy of three groups

There were no significant differences in recanalization rate, sICH, asymptomatic intracranial hemorrhage transformation, mRS, mortality and poor outcome among the three groups (*P*>0.05). The cost in the DD group was lower than that in the MS and DS groups (64258 vs 80041 vs 70750, *P*=0.018) (Table 2).

**Table 2:** Outcomes after endovascular thrombectomy of three referral modes

**Outcomes**	***DD*** ***N=83***	**MS** ***N*=85**	**DS** ***N*=181**	***P*-value**
**Recanalization(n,%)**	68 (81.93%)	74 (87.06%)	143 (79.01%)	0.285
**Cost, M(IQR)**	64258.00 (45832.00-88586.50)	80041.00 (59036.00-110982.00)	70750.00 (52046.00-92235.00)	0.018
**sICH(n,%)**	19 (22.89%)	16 (18.82%)	35 (19.34%)	0.758
**asICH(n,%)**	19 (22.89%)	26 (30.59%)	38 (20.99%)	0.225
**mRS, M(IQR)**	4.00 (2.00-5.00)	4.00 (1.00-5.00)	4.00 (2.00-6.00)	0.836
**mRS(n,%)**				0.072
**0**	3 (3.61%)	6 (7.06%)	11 (6.08%)	
**1**	10 (12.05%)	16 (18.82%)	32 (17.68%)	
**2**	14 (16.87%)	8 (9.41%)	14 (7.73%)	
**3**	9 (10.84%)	7 (8.24%)	28 (15.47%)	
**4**	15 (18.07%)	19 (22.35%)	35 (19.34%)	
**5**	12 (14.46%)	8 (9.41%)	7 (3.87%)	
**6**	20 (24.10%)	21 (24.71%)	54 (29.83%)	
**Mortality(n,%)**	20 (24.10%)	21 (24.71%)	54 (29.83%)	0.521
**Poor outcome(n,%)**	56 (64.47%)	55 (64.71%)	124 (68.51%)	0.827

*M* Median, *IQR* Interquartile range, *DD* Drive the Doctor, *MS* Mothership, *DS* Drip and Ship, *sICH* Symptomatic intracranial hemorrhage transformation, *asICH* Asymptomatic intracranial hemorrhage transformation, *mRS* Modified Rankin Scale.

#### Time to treatment

There were significant statistical differences in ODT, OPT, PRT, DPT and DRT among the three groups. Compared with MS and DS group, the ODT and OPT of DD group were shorter (90min vs 166min vs 170min, *P*<0.001; 230mm vs 270min vs 270min, *P*<0.001), PRT, DPT and DRT were longer (82min vs 54min vs 51min, *P*<0.001; 110min vs 85min vs 96min, *P*=0.004; 210min vs 146min vs 150min, *P*<0.001). In PRT, DS group is the shortest and in DPT, DRT, MS group is the shortest. There was no statistical difference in ORT among the three groups (305min vs 328min vs 335min, *P*=0.260) (Table 3).

**Table 3:** Operation-related time indicators of three referral modes

**Time(min)**	**DD** ***N*=83**	**MS** ***N*=85**	**DS** ***N*=181**	***P*-value**
**ODT, M(IQR)**	90.00 (56.00-144.00)	166.00 (107.00-276.00)	170.00 (112.82-260.00)	<0.001
**OPT, M(IQR)**	230.00 (172.50-289.00)	270.00 (220.00-360.00)	270.00 (212.00-365.00)	<0.001
**ORT, M(IQR)**	305.00 (251.50-381.00)	328.00 (280.00-405.00)	335.00 (265.00-435.00)	0.260
**PRT, M(IQR)**	82.00 (60.00-110.00)	54.00 (39.00-82.00)	51.00 (35.00-75.00)	<0.001
**DPT, M(IQR)**	110.00 (74.50-173.00)	85.00 (55.00-134.00)	96.00 (50.00-143.89)	0.004
**DRT, M(IQR)**	210.00 (159.00-278.50)	146.00 (119.00-191.00)	150.00 (111.00-202.61)	<0.001

*M* Median, *IQR* Interquartile range, *DD* Drive the Doctor, *MS* Mothership, *DS* Drip and Ship, *ODT* Onset-to-door time, *OPT* Onset-to-puncture time *ORT* Onset-to-recanalization time, *PRT* Puncture-to-recanalization time, *DPT* Door-to-puncture time, *DRT* Door-to-recanalization time.

### Multiple regression analysis of referral modes among three groups

Compared with DD group, there was no independent correlation between different referral modes in sICH, poor outcome and mortality (*P*>0.05). In the unadjusted model, the MS group was independently associated with the cost (*β*=20529.94, 95%CI:3204.23-37855.65, *P*=0.0208). After adjusting for the age and gender in the Model I, the MS group was still independently associated with the cost (*β*=20535.56, 95%CI:3122.93-37948.20, *P*=0.0214). After adjusting for the variables with statistical differences and related covariables in the univariate analysis in the Model II, there was a significant correlation between the MS group and the cost (*β*=30449.73, 95%CI:11022.18-49877.29, *P*=0.0023). The cost of MS group was much higher than that of DD group (Table 4).

**Table 4:** Multiple regression analysis of referral modes among the three groups

	**Unadjusted**	**Model I**	**Model II**
	***OR*/*β*(95%CI) *P***	***OR*/*β*(95%CI) *P***	***OR*/*β*(95%CI) *P***
**sICH**			
**Mode**			
**DD**	1.0	1.0	1.0
**MS**	0.78 (0.37, 1.65) 0.5168	0.77 (0.36, 1.64) 0.5015	0.50 (0.18, 1.38) 0.1830
**DS**	0.81 (0.43, 1.52) 0.5067	0.83 (0.44, 1.56) 0.5530	0.47 (0.19, 1.16) 0.1000
**Poor outcome**			
**Mode**			
**DD**	1.0	1.0	1.0
**MS**	0.88 (0.47, 1.68) 0.7053	0.87 (0.45, 1.67) 0.6789	0.61 (0.25, 1.47) 0.2705
**DS**	1.05 (0.60, 1.83) 0.8664	1.13 (0.64, 1.99) 0.6794	0.53 (0.24, 1.18) 0.1223
**Mortality**			
**Mode**			
**DD**	1.0	1.0	1.0
**MS**	1.03 (0.51, 2.09) 0.9267	1.05 (0.51, 2.15) 0.8886	0.56 (0.19, 1.67) 0.2993
**DS**	1.34 (0.74, 2.43) 0.3361	1.44 (0.79, 2.65) 0.2372	0.65 (0.25, 1.69) 0.3744
**Cost**			
**Mode**			
**DD**	0	0	0
**MS**	20529.94 (3204.23, 37855.65) 0.0208	20535.56 (3122.93, 37948.20) 0.0214	30449.73 (11022.18, 49877.29) 0.0023
**DS**	5834.54 (-9049.08, 20718.16) 0.4428	5910.74 (-9056.35, 20877.82) 0.4394	14630.17 (-2428.95, 31689.30) 0.0938

Model I: adjusted for age and gender; Model II: adjusted the variables with statistical differences in univariate analysis Table S5, 6, 7 and 8 (*P*<0.1) and the covariates selected in Table S14, 15, 16, and 17 in the appendix; *sICH* Symptomatic intracranial hemorrhage transformation, *DD* Drive the Doctor, *MS* Mothership, *DS* Drip and Ship.

### Stratified analysis of referral modes among the three groups

According to different outcome variables, further stratified analysis was made according to age (< 65 years old vs ≥ 65 years old), gender (male vs female), related risk factors (no vs yes), intravenous thrombolysis (no vs yes) and NIHSS (< 16 vs ≥ 16).

The results of sICH showed that compared with DD group, the influences of different referral modes on sICH were significantly different in age groups. Among the patients younger than 65 years old, MS group had significantly higher risks of sICH (*OR*=29.88, 95%CI:1.28-696.19, *P*=0.0345), the risk of sICH in MS group was 28.88 times higher than that in DD group. In patients older than 65 years old, MS and DS groups had lower risks of sICH (*OR*=0.15, 95%CI:0.04-0.59, *P*=0.0067; *OR*=0.27, 95%CI:0.08-0.88, *P*=0.0304), the risk of sICH was reduced by 85% and 73% compared with DD group, respectively. In addition, in patients without hyperlipidemia, the risk of sICH in the DS group was 76% lower than that in the DD group (*OR*=0.24, 95%CI:0.07-0.79, *P*=0.0197). The remaining indexes were not statistically significant (Table S1).

The results of poor outcome showed that there were significant differences in the impact of different referral modes on poor outcome in gender compared with DD group. In male patients, the DS group was negatively correlated with poor outcome, with a 67% lower risk of poor outcome than DD group (*OR*=0.33, 95%CI:0.11-0.97, *P*=0.0436). The other indexes were not statistically significant (Table S2).

The results of mortality showed that with reference to DD group, different referral modes were negatively correlated with mortality in patients without hyperlipidemia. The risk of death in MS group and DS group was 81% and 74% lower than that in DD group, respectively (*OR*=0.19, 95%CI:0.04-0.81, *P*=0.0248; *OR*=0.26, 95%CI:0.07-0.92, *P*=0.0370). The other indexes were not statistically significant (Table S3).

The results of cost showed that in DD group, there were significant differences in age, gender, risk factors, intravenous thrombolysis and NIHSS. Compared with DD group, MS group spent more money than DD group in male patients, patients with hypertension, without atrial fibrillation, without coronary heart disease, without hyperlipidemia, without previous stroke, without smoking history, or without intravenous thrombolysis (*P*<0.05). The cost in MS group was higher than that in DD group regardless of age and NIHSS (*P*<0.05). Compared with DD group, the cost of DS group was higher than DD group in male patients, patients without atrial fibrillation, or without intravenous thrombolysis (*P*<0.05), that is, the cost advantage of DD group was more obvious (Table S4).

### Multiple regression analysis of group A and group B

Table S20 summarized the results that there was no correlation regarding sICH, poor outcome, mortality or cost between groups in the unadjusted model (*P*=0.4607; *P*=0.9761; *P*=0.4644; *P*=0.1462). After adjusting gender and age in model I, the results were still not correlated (*P*=0.4862; *P*=0.8975; *P*=0.3657; *P*=0.1459). However, after adjusting variables with statistical differences in univariate analysis and related covariates in model II, group B was independently associated with cost (*β*=-19438.86, 95%CI: -35977.79- -2899.94, *P*=0.0219). Patients paid significantly less for endovascular therapy at a PSC than at a CSC (Table S20).

## Discussion

Our study found that DD mode was more economical, with no significant difference in sICH, mortality and poor outcome after EVT, compared to MS and DS modes. It suggested that DD mode was an alternative for MS and DS modes which are majority referral modes in some extent. These results would be interpreted in the context of 3 following debates.

### Analysis of different referral modes and outcomes

Hemorrhagic transformation is a complication of acute ischemic stroke, resulting in poor outcomes and mortality [27]. In our study, there was no significant difference in sICH among the three groups (*P*=0.758). Multiple regression analysis found that there was no independent correlation between the three referral modes and sICH (*P*>0.05), which was consistent with the results of Kijpaisalratana et al. [28]. Subsequently, we made a further stratified analysis of sICH in different subgroups. The results showed MS mode was more likely to develop sICH in patients less than 65 years old, which may be related to the longer delay of first admission of stroke patients in MS mode than in the other two modes. Earlier admission should reduce the risk of sICH after endovascular therapy by controlling risk factor earlier. Unlike in MS and DS modes, patients 65 years or older in DD mode have a higher risk of sICH because the perioperative management experience of the PSC are lower than those of the CSC, even if endovascular therapy is performed by experienced neurointerventional surgeons. In addition, age is an unchangeable risk factor for stroke. The intracranial vascular conditions of elderly patients are worse, and their tolerance to changes in blood pressure is poorer. Related studies have shown that patients with elevated blood pressure and high blood pressure variability have an increased risk of hemorrhagic transformation, [29] so the risk of hemorrhagic transformation in elderly patients is higher than that in young people. This situation can be improved by improving the inter-hospital cooperation mechanism and strengthening the training of medical staff in PSC on the perioperative management of EVT.

The related study shows that for per 1 hour delay in the onset and recanalization time of patients with acute ischemic stroke, the probability of functional independence is reduced by 10%-38% [13, 17]. There was no significant difference in poor outcome and mortality among the three groups in this study (*P*=0.827, *P*=0.521), and multiple regression analysis still showed that there was no independent correlation between different referral modes in poor outcome and mortality (*P*>0.05), which was similar to that of Seker et al and Kijpaisalratana et al. [15, 28]. It is proved that the clinical outcomes of patients with DD mode is as good as that of MS and DS modes, which are more mature. Further stratified analysis of poor outcome showed that in male patients, the risk of poor outcome in DS mode was lower than that in DD mode (*OR*=0.33, 95%CI:0.11-0.97, *P*=0.0436). In general, cardiac embolism (CE) stroke was more common in females and large artery atherosclerosis (LAA) thrombotic stroke was more common in males [30, 31]. LAA lesions may result in intracranial atherosclerosis (ICAS), and form ICAS-related occlusions (ICAS-O) easily. Related studies indicated that current techniques of stent-retriever and aspiration thrombectomy are highly effective in CE stroke. However, these techniques are less efficacious in ICAS-O stroke and successful recanalization of ICAS-O may require rescue treatments with intra-arterial thrombolysis, balloon angioplasty or stent placement [32, 33]. In our study, the rate of LAA (117/208, 56.25%) in males was higher than CE (76/208, 36.54%) and other/undetermined etiology (15/208, 7.21%). Totally seventy-eight male patients, forty in DS group and twenty-four in DD group, received rescue treatments. We speculated that more male patients received rescue treatments in DS group compared to the male patients in DD group because of the alternatives of techniques such as balloon or stent in CSC were more than that in PSC. Besides, postoperative nursing and complication management in CSC were superior to that in PSC, which influenced the recovery of male patients in some extent. Therefore, the risk of poor outcome in male patients was lower in DS group for the variety of techniques and experienced management in CSC. However, the relative study shows that there is no significant difference in outcomes between DS mode and DD mode [19], and the results be caused by the fact that most patients of the three groups are male. Related studies have shown that women are independent risk factors for poor outcome and impaired quality of life of ischemic stroke. The prevalence rate of stroke in women is higher, and the risk of stroke increases with age [34]. This requires further study after including more female stroke patients who meet the criteria.

In addition, according to the stratified analysis of different referral modes and mortality, we found that in patients without hyperlipidemia, the risk of death in DD mode was higher than that in MS and DS mode (*OR*=0.19, 95%CI:0.04-0.81, *P*=0.0248; *OR*=0.26, 95%CI:0.07-0.92, *P*=0.0370). Due to the lack of similar results reported in previous studies, further studies are needed to explain this result.

Finally, our study also chooses cost as the outcome to explore the differences among the three clinical referral modes in a view of economic. Table 2 showed that the cost of DD mode was less than that of MS mode and DS mode, and there was a statistical difference (*P*=0.018). Multiple regression analysis showed that the cost of DD mode was less than that of MS mode (*β*=30449.73, 95% CI:11022.18-49877.29, *P*=0.0023). DD mode greatly reduces the economic expenditure of stroke patients' families, which means it would become a better substitute for MS and DS modes. The reduction in treatment costs probably because of inconsistencies in treatment-related costs such as auxiliary examinations, treatment drugs, stroke care, etc. between PSC and CSC at different grades hospitals. Further stratified analysis of cost also shows that the cost of DD mode is less than that of MS and DS modes in most cases, resulting in huge social and economic benefits.

Furthermore, we divided the patients into two groups that finally admitted to different stroke centers, and multiple regression analysis showed a more obvious cost advantage of DD mode (Table S20). At present, study on the cost of different referral modes is lacked. Kunz et al.'s results of endovascular therapy for patients with ischemic stroke showed that the incremental cost-effectiveness ratio (ICER) of quality-adjusted life year (QALY) generated by endovascular therapy plus standard care costs for stroke was $22801 [35]. Related research by Simpson et al shows that the average cost of stroke patients receiving endovascular therapy abroad is $35130 [36]. Barbosa et al's study suggests that the cost-effectiveness of intravenous thrombolysis and endovascular therapy in three developing countries (Iran, China and Brazil) is about $2578 to $34052 per QALY, of which the cost-effectiveness of each QALY in China (based on the exchange rate between China and the United States in 2019) is $10142 [37]. The cost result of our study is similar to Barbosa et al's, [37] but it is still far less than the cost of endovascular therapy for stroke patients abroad.

### Intravenous thrombolysis among the three referral modes

The proportion of intravenous thrombolysis in DD group was higher than that in MS group and DS group (49 (59.04%) vs 30 (35.29%) vs 30(33.15%), *P*<0.001) of our study, which is similar to the proportion of intravenous thrombolysis (63.5%, 32.4%, 59.8%) in Seker et al. [15]. The difference in the DS group probably due to the fact that once the PSC judge that patients with acute ischemic stroke may have LVO, they tended to refer the patients directly to the CSC in order to minimize the delay of secondary transport between hospitals as much as possible. When the patients arrive at the CSC, they may have exceeded the time window of intravenous thrombolysis.

### Time indicators analysis among the three referral modes

The relationships between the establishment of reperfusion and outcomes are time-dependent [38, 39]. In this study, there were statistical differences in most of the time indicators among the three groups of modes.

The ODT and OPT in DD group were shorter than those in MS group and DS group (*P*<0.001, *P*<0.001). This may be on account of the location of endovascular therapy in DD group is located in the PSC, which avoids the second referral between different hospitals, and transferring neurointerventional surgeons rather than patients costs less time. The physicians shortened the OPT through advanced preoperative preparation, including femoral artery puncture before the neurointerventional surgeons arrived at the PSC. Among the results of Seker et al, [15] the OPT of MS mode was the shortest, the OPT of DD mode was in the middle and the OPT of DS mode was the longest. The results of Ismail et al also showed that the OPT of MS mode was the shortest, [16] which contradicts to our study. The reason may be that all the PSCs in this study have junior doctors engaged in neurointervention who are qualified to perform percutaneous femoral artery catheterization, although lack of skilled endovascular therapy experience, can quickly complete preoperative preparation.

The PRT, DPT and DRT of DD mode were longer than those of MS mode and DS mode (*P*<0.001, *P*=0.004, *P*<0.001), which is similar to the results of Wei et al. [17] The prolongation of PRT and DRT may be caused by the available number of CSC is limited to patients and the workload in CSC is heavy, in addition to that, most CSC are usually concentrated in urban areas [40]. The waiting time for the arrival of neurointerventional surgeons of CSC to formally start endovascular therapy after femoral artery puncture prolongs the overall time of operation and delays the recanalization time. This condition may be improved with the increase in the overall number of CSC. The PRT in Multi-MERCI study is 96 minutes (IQR, 18-282min) [41]. Taking this as a reference, although the DD mode in this study extends the patient's PRT to some extent, it is shorter than the Multi-MERCI study (82min vs 96min), which may be attributed to the progress of medical technology and devices and experienced neurointerventional surgeons. With the standardization and normalization of neurovascular interventional therapy, PRT and DRT will be further shortened. Wei et al showed that the DPT of DD mode was 128min, [17] and that of DD mode in our study was 110min, which is similar. The CSC in the MS mode has more experience in handling the first aid flow in the stroke patients than the PSC in the DD mode, and PSCs in the DS mode contacted the CSC in advance before patients were referred to the CSC for avoiding unnecessary delay after re-admission, decreasing the time consumed in DPT. Because of above reasons, DPT in the DD mode of our study is more time-consuming than MS and DS modes.

Compared with the results of Osanai et al, [18] the operation-related time indicators in this study are longer, which may be due to the relatively late start of neurointerventional therapy in our country and the accumulation of pre-hospital and in-hospital time delay caused by immature procedures. This will be improved after the endovascular therapy gradually matures.

Our study has some advantages: First, although this study was a retrospective study, which inevitably included many potential confounding factors, we used strict statistical analysis as much as possible to minimize the influences of confounding factors. Second, the results of stratified analysis suggested that DD mode may increase the risk of sICH in patients older than 65 years old, but greatly reduce the related costs of stroke treatments. Third, our study provided a reference for the choice of referral modes in clinical work. But the shortcomings of this study are also obvious: First, the retrospective study can only provide weak evidence of the correlation between exposure variables and outcome variables, and it was difficult to distinguish their causality. Second, the research group was limited in the regions with economic advantages and convenient transportation, therefore it can’t be extended to other regions temporarily. Third, the application of DD mode is not mature enough and needs further promotion.

## Conclusion

There was no independent correlation between three referral modes and sICH, mortality, poor outcome correspondingly. Different referral modes can be implemented in clinical practice according to the situations encountered. Considering the cost, compared with MS and DS mode, DD mode is more economical. 

## Supplementary Information

**Additional file 1:** **Table S1. **Stratified Analysis of sICH among three modes. **Table S2. **Stratified Analysis of poor outcome among three modes. **Table S3. **Stratified Analysis of mortality among three modes. **Table S4. **Stratified Analysis of cost among three modes. **Table S5. **Univariate regression Analysis of sICH. **Table S6. **Univariate regression Analysis of poor outcome. **TableS7. **Univariate regression Analysis of mortality. **Table S8. **Univariate regression Analysis of cost. **Table S9. **Collinear screening. **Table S10. **The relationship between covariates and the sICH (*n*=349). **Table S11. **The relationship between covariates and poor outcome (*n*=349). **Table S12. **The relationship between covariates and mortality (*n*=349). **Table S13. **The relationship between covariates and cost (*n*=349).**Table S14. **The adjusting effect of potential mixed factors on the estimated value of sICH. **Table S15. **The adjusting effect of potential mixed factors on the estimated value of poor outcome. **Table S16.**The adjusting effect of potential mixed factors on the estimated value of mortality. **Table S17. **The adjusting effect of potential mixed factors on the estimated value of cost. **Table S18. **Baseline data of group A and B. **Table S19.** Outcomes after endovascular thrombectomy of group A and B. **Table S20. **Multiple regression analysis of group A and B. 

## Data Availability

The datasets generated and/or analysed during the current study are not publicly available due to the datasets are owned by the institution only but are available from the corresponding author on reasonable request.
