# Supplementary material for: Influences of different referral modes on clinical outcomes after endovascular therapy for acute ischemic stroke
Source: BMC Neurol. 2022 Jun 21;22:228. doi: 10.1186/s12883-022-02751-w (PMC9210676; doi:10.1186/s12883-022-02751-w)
Supplement: Supplementary file 1 — Additional file 1: Table S1. Stratified Analysis of sICH among three modes. Table S2. Stratified Analysis of poor outcome among three modes. Table S3. Stratified Analysis of mortality among three modes. Table S4. Stratified Analysis of cost among three modes. Table S5. Univariate regression Analysis of sICH. Table S6. Univariate regression Analysis of poor outcome. TableS7. Univariate regression Analysis of mortality. Table S8. Univariate regression Analysis of cost. Table S9. Collinear screening. Table S10. The relationship between covariates and the sICH (n=349). Table S11. The relationship between covariates and poor outcome (n=349). Table S12. The relationship between covariates and mortality (n=349). Table S13. The relationship between covariates and cost (n=349).Table S14. The adjusting effect of potential mixed factors on the estimated value of sICH. Table S15. The adjusting effect of potential mixed factors on the estimated value of poor outcome. Table S16.The adjusting effect of potential mixed factors on the estimated value of mortality. Table S17. The adjusting effect of potential mixed factors on the estimated value of cost. Table S18. Baseline data of group A and B. Table S19. Outcomes after endovascular thrombectomy of group A and B. Table S20. Multiple regression analysis of group A and B. [file 12883_2022_2751_MOESM1_ESM.docx]

[**Supplymentary**](javascript:;) **materials**

**Influences of different referral modes on clinical outcomes after endovascular therapy for acute ischemic stroke**

**Table S1 Stratified Analysis of sICH among three modes**

|  | **DD**  **Ref** | **MS**  ***OR*(95%CI) *P*** | **DS**  ***OR*(95%CI) *P*** |
| --- | --- | --- | --- |
| **Age** |  |  |  |
| **<65** | 1.0 | 29.88 (1.28, 696.19) 0.0345 | 6.22 (0.45, 86.42) 0.1737 |
| **≥65** | 1.0 | 0.15 (0.04, 0.59) 0.0067 | 0.27 (0.08, 0.88) 0.0304 |
| **Gender** |  |  |  |
| **male** | 1.0 | 0.80 (0.18, 3.49) 0.7625 | 0.56 (0.16, 1.98) 0.3705 |
| **female** | 1.0 | 0.19 (0.03, 1.22) 0.0796 | 0.48 (0.07, 3.35) 0.4564 |
| **Hypertension** |  |  |  |
| **no** | 1.0 | 0.07 (0.00, 1.31) 0.0755 | 0.55 (0.08, 3.76) 0.5439 |
| **yes** | 1.0 | 0.73 (0.21, 2.60) 0.6277 | 0.48 (0.15, 1.57) 0.2247 |
| [**Atrial**](javascript:;) [**fibrillation**](javascript:;) |  |  |  |
| **no** | 1.0 | 0.65 (0.13, 3.41) 0.6136 | 0.29 (0.06, 1.30) 0.1059 |
| **yes** | 1.0 | 0.23 (0.04, 1.40) 0.1104 | 0.31 (0.06, 1.64) 0.1677 |
| [**Coronary**](javascript:;) [**heart**](javascript:;) [**disease**](javascript:;) |  |  |  |
| **no** | 1.0 | 0.39 (0.13, 1.17) 0.0948 | 0.44 (0.17, 1.15) 0.0935 |
| **yes** | 1.0 | inf^a^. (0.00, Inf^a^) 0.9999 | inf^a^. (0.00, Inf^a^) 0.9999 |
| [**Hyperlipemia**](javascript:;) |  |  |  |
| **no** | 1.0 | 0.28 (0.07, 1.03) 0.0548 | 0.24 (0.07, 0.79) 0.0197 |
| **yes** | 1.0 | 1.06 (0.06, 20.35) 0.9698 | 1.11 (0.08, 15.15) 0.9392 |
| **Previous stroke** |  |  |  |
| **no** | 1.0 | 0.45 (0.14, 1.48) 0.1907 | 0.37 (0.12, 1.11) 0.0759 |
| **yes** | 1.0 | 0.00 (0.00, Inf^a^) 0.9997 | 0.00 (0.00, Inf^a^) 0.9999 |
| **Smoking** |  |  |  |
| **no** | 1.0 | 0.63 (0.19, 2.04) 0.4366 | 0.65 (0.22, 1.87) 0.4205 |
| **yes** | 1.0 | 0.00 (0.00, Inf^a^) 0.9997 | 0.00 (0.00, Inf^a^) 0.9996 |
| [**Intravenous**](javascript:;) [**thrombolysis**](javascript:;) |  |  |  |
| **no** | 1.0 | 0.35 (0.08, 1.67) 0.1904 | 0.41 (0.10, 1.63) 0.2035 |
| **yes** | 1.0 | 0.91 (0.05, 15.57) 0.9472 | 0.69 (0.07, 7.17) 0.7555 |
| **NIHSS** |  |  |  |
| **<16** | 1.0 | 3.94 (0.22, 69.67) 0.3499 | 0.39 (0.04, 3.71) 0.4103 |
| **≥16** | 1.0 | 0.33 (0.09, 1.27) 0.1065 | 0.51 (0.15, 1.70) 0.2709 |

Ref: Reference; included age, gender, hypertension, atrial fibrillation, hyperlipidemia, intravenous thrombolysis, NIHSS and other confounding factors, unless the included variables are stratified variables; sICH: Symptomatic intracranial hemorrhage transformation; DD: Drive the Doctor; MS: Mothership; DS: Drip and Ship; NIHSS: National Institutes of Health Stroke Scale.

^a^ Due to the small sample size, the model analysis failed.

**Table S2 Stratified Analysis of poor outcome among three modes**

|  | **DD**  **Ref** | **MS**  ***OR(*95%CI) *P*** | **DS**  ***OR*(95%CI) *P*** |
| --- | --- | --- | --- |
| **Age** |  |  |  |
| **<65** | 1.0 | 0.58 (0.10, 3.36) 0.5399 | 0.22 (0.04, 1.15) 0.0733 |
| **≥65** | 1.0 | 0.64 (0.18, 2.24) 0.4812 | 0.78 (0.24, 2.49) 0.6735 |
| **Gender** |  |  |  |
| **male** | 1.0 | 0.36 (0.10, 1.30) 0.1190 | 0.33 (0.11, 0.97) 0.0436 |
| **female** | 1.0 | 1.99 (0.33, 12.03) 0.4544 | 1.50 (0.26, 8.76) 0.6499 |
| **Hypertension** |  |  |  |
| **no** | 1.0 | 0.09 (0.01, 1.17) 0.0657 | 0.70 (0.06, 8.00) 0.7767 |
| **yes** | 1.0 | 1.08 (0.35, 3.31) 0.8944 | 0.63 (0.24, 1.67) 0.3507 |
| [**Atrial**](javascript:;) [**fibrillation**](javascript:;) |  |  |  |
| **no** | 1.0 | 0.72 (0.20, 2.57) 0.6161 | 0.72 (0.23, 2.25) 0.5700 |
| **yes** | 1.0 | 0.72 (0.16, 3.32) 0.6761 | 0.55 (0.12, 2.50) 0.4403 |
| [**Coronary**](javascript:;) [**heart**](javascript:;) [**disease**](javascript:;) |  |  |  |
| **no** | 1.0 | 0.76 (0.30, 1.90) 0.5532 | 0.50 (0.22, 1.15) 0.1032 |
| **yes** | 1.0 | inf^a^. (0.00, Inf^a^) 1.0000 | inf^a^. (0.00, Inf^a^) 1.0000 |
| [**Hyperlipemia**](javascript:;) |  |  |  |
| **no** | 1.0 | 0.53 (0.18, 1.56) 0.2485 | 0.54 (0.19, 1.52) 0.2453 |
| **yes** | 1.0 | 3.09 (0.19, 50.27) 0.4273 | 1.41 (0.20, 10.15) 0.7348 |
| **Previous stroke** |  |  |  |
| **no** | 1.0 | 0.46 (0.17, 1.26) 0.1311 | 0.50 (0.19, 1.27) 0.1426 |
| **yes** | 1.0 | inf^a^. (0.00, Inf^a^) 0.9998 | 0.00 (0.00, Inf^a^) 0.9999 |
| **Smoking** |  |  |  |
| **no** | 1.0 | 1.01 (0.34, 3.03) 0.9850 | 1.07 (0.38, 2.99) 0.8967 |
| **yes** | 1.0 | 0.40 (0.04, 3.78) 0.4262 | 0.17 (0.02, 1.34) 0.0918 |
| [**Intravenous**](javascript:;) [**thrombolysis**](javascript:;) |  |  |  |
| **no** | 1.0 | 0.58 (0.16, 2.20) 0.4261 | 0.62 (0.20, 1.95) 0.4130 |
| **yes** | 1.0 | 0.57 (0.12, 2.80) 0.4878 | 0.23 (0.05, 1.10) 0.0664 |
| **NIHSS** |  |  |  |
| **<16** | 1.0 | 0.68 (0.20, 2.33) 0.5356 | 0.98 (0.34, 2.85) 0.9747 |
| **≥16** | 1.0 | 0.56 (0.12, 2.50) 0.4458 | 0.45 (0.12, 1.74) 0.2462 |

Ref: Reference; included age, gender, hypertension, atrial fibrillation, hyperlipidemia, intravenous thrombolysis, NIHSS and other confounding factors, unless the included variables are stratified variables; DD: Drive the Doctor; MS: Mothership; DS: Drip and Ship; NIHSS: National Institutes of Health Stroke Scale.

^a^ Due to the small sample size, the model analysis failed.

**Table S3 Stratified Analysis of mortality among three modes**

|  | **DD**  **Ref** | **MS**  ***OR*(95%CI) *P*** | **DS**  ***OR*(95%CI) *P*** |
| --- | --- | --- | --- |
| **Age** |  |  |  |
| **<65** | 1.0 | 0.11 (0.00, 9.97) 0.3396 | 0.07 (0.00, 3.30) 0.1793 |
| **≥65** | 1.0 | 0.45 (0.11, 1.74) 0.2463 | 0.60 (0.18, 2.02) 0.4145 |
| **Gender** |  |  |  |
| **male** | 1.0 | 0.57 (0.11, 3.03) 0.5066 | 0.89 (0.21, 3.73) 0.8697 |
| **female** | 1.0 | 0.26 (0.03, 2.11) 0.2083 | 1.02 (0.13, 8.06) 0.9841 |
| **Hypertension** |  |  |  |
| **no** | 1.0 | 0.15 (0.00, 6.29) 0.3232 | 0.20 (0.01, 3.78) 0.2840 |
| **yes** | 1.0 | 0.71 (0.17, 3.01) 0.6393 | 1.10 (0.31, 3.92) 0.8780 |
| [**Atrial**](javascript:;) [**fibrillation**](javascript:;) |  |  |  |
| **no** | 1.0 | 0.36 (0.06, 1.97) 0.2383 | 0.75 (0.19, 2.96) 0.6800 |
| **yes** | 1.0 | 0.38 (0.05, 3.05) 0.3635 | 0.35 (0.05, 2.37) 0.2838 |
| [**Coronary**](javascript:;) [**heart**](javascript:;) [**disease**](javascript:;) |  |  |  |
| **no** | 1.0 | 0.77 (0.23, 2.56) 0.6716 | 0.98 (0.34, 2.81) 0.9681 |
| **yes** | 1.0 | inf^a^. (0.00, Inf^a^) 0.9999 | inf^a^. (0.00, Inf^a^) 1.0000 |
| [**Hyperlipemia**](javascript:;) |  |  |  |
| **no** | 1.0 | 0.19 (0.04, 0.81) 0.0248 | 0.26 (0.07, 0.92) 0.0370 |
| **yes** | 1.0 | 5.64 (0.24, 133.82) 0.2841 | 10.56 (0.56, 198.25) 0.1152 |
| **Previous stroke** |  |  |  |
| **no** | 1.0 | 0.62 (0.15, 2.53) 0.5076 | 0.81 (0.22, 2.91) 0.7438 |
| **yes** | 1.0 | 0.07 (0.00, 20.84) 0.3571 | 1.16 (0.01, 143.31) 0.9513 |
| **Smoking** |  |  |  |
| **no** | 1.0 | 0.54 (0.14, 2.06) 0.3653 | 0.88 (0.28, 2.75) 0.8251 |
| **yes** | 1.0 | 0.00 (0.00, Inf^a^) 0.9999 | 0.00 (0.00, Inf^a^) 0.9994 |
| [**Intravenous**](javascript:;) [**thrombolysis**](javascript:;) |  |  |  |
| **no** | 1.0 | 0.54 (0.11, 2.57) 0.4392 | 0.72 (0.19, 2.78) 0.6350 |
| **yes** | 1.0 | 0.18 (0.01, 5.41) 0.3239 | 0.25 (0.02, 3.64) 0.3138 |
| **NIHSS** |  |  |  |
| **<16** | 1.0 | 0.68 (0.20, 2.33) 0.5356 | 0.98 (0.34, 2.85) 0.9747 |
| **≥16** | 1.0 | 0.56 (0.12, 2.50) 0.4458 | 0.45 (0.12, 1.74) 0.2462 |

Ref: Reference; included age, gender, hypertension, atrial fibrillation, hyperlipidemia, intravenous thrombolysis, NIHSS and other confounding factors, unless the included variables are stratified variables; DD: Drive the Doctor; MS: Mothership; DS: Drip and Ship; NIHSS: National Institutes of Health Stroke Scale.

^a^ Due to the small sample size, the model analysis failed.

**Table S4 Stratified Analysis of cost among three modes**

|  | **DD**  **Ref** | **MS**  ***β*(95%CI) *P*** | **DS**  ***β*(95%CI) *P*** |
| --- | --- | --- | --- |
| **Age** |  |  |  |
| **<65** | 0 | 32640.20 (6044.73, 59235.66) 0.0179 | 11998.90 (-10332.08, 34329.88) 0.2946 |
| **≥65** | 0 | 31136.93 (2245.81, 60028.06) 0.0361 | 21249.93 (-5280.33, 47780.19) 0.1183 |
| **Gender** |  |  |  |
| **male** | 0 | 32900.96 (12156.14, 53645.79) 0.0022 | 20011.46 (3118.38, 36904.54) 0.0214 |
| **female** | 0 | 28131.92 (-10571.59, 66835.43) 0.1572 | 1472.91 (-36524.35, 39470.17) 0.9396 |
| **Hypertension** |  |  |  |
| **no** | 0 | 12870.40 (-17507.37, 43248.16) 0.4088 | -761.75 (-27352.30, 25828.81) 0.9554 |
| **yes** | 0 | 35345.91 (9111.18, 61580.63) 0.0089 | 20893.09 (-1882.16, 43668.33) 0.0737 |
| [**Atrial**](javascript:;) [**fibrillation**](javascript:;) |  |  |  |
| **no** | 0 | 36398.25 (6767.27, 66029.22) 0.0172 | 33173.45 (8057.14, 58289.75) 0.0105 |
| **yes** | 0 | 20012.29 (-6411.09, 46435.67) 0.1404 | -13648.22 (-37929.89, 10633.44) 0.2729 |
| [**Coronary**](javascript:;) [**heart**](javascript:;) [**disease**](javascript:;) |  |  |  |
| **no** | 0 | 28093.58 (7237.77, 48949.39) 0.0087 | 14249.69 (-4169.77, 32669.15) 0.1306 |
| **yes** | 0 | -10176.40 (, ) NaN^a^ | 48536.92 (, ) NaN^a^ |
| [**Hyperlipemia**](javascript:;) |  |  |  |
| **no** | 0 | 27654.28 (8925.09, 46383.46) 0.0042 | 13079.45 (-4135.56, 30294.46) 0.1380 |
| **yes** | 0 | 20077.54 (-39357.58, 79512.65) 0.5098 | 13500.25 (-34706.70, 61707.20) 0.5846 |
| **Previous stroke** |  |  |  |
| **no** | 0 | 31106.31 (8028.92, 54183.69) 0.0088 | 18197.44 (-2581.58, 38976.46) 0.0873 |
| **yes** | 0 | 36963.66 (-7457.38, 81384.69) 0.1114 | -789.82 (-40584.46, 39004.82) 0.9692 |
| **Smoking** |  |  |  |
| **no** | 0 | 37097.65 (11428.24, 62767.07) 0.0051 | 18004.73 (-4810.71, 40820.17) 0.1234 |
| **yes** | 0 | 20883.78 (-4794.23, 46561.79) 0.1157 | 11712.88 (-10434.96, 33860.72) 0.3037 |
| [**Intravenous**](javascript:;) [**thrombolysis**](javascript:;) |  |  |  |
| **no** | 0 | 38369.83 (17819.35, 58920.31) 0.0003 | 18488.49 (388.98, 36588.00) 0.0468 |
| **yes** | 0 | 22326.08 (-19784.04, 64436.20) 0.3011 | 7080.92 (-29854.89, 44016.72) 0.7079 |
| **NIHSS** |  |  |  |
| **<16** | 0 | 23082.01 (249.67, 45914.35) 0.0495 | 8950.84 (-10358.26, 28259.94) 0.3651 |
| **≥16** | 0 | 39390.81 (4431.18, 74350.43) 0.0288 | 24147.04 (-7376.77, 55670.85) 0.1355 |

Ref: Reference; included age, gender, hypertension, atrial fibrillation, hyperlipidemia, intravenous thrombolysis, NIHSS and other confounding factors, unless the included variables are stratified variables; DD: Drive the Doctor; MS: Mothership; DS: Drip and Ship; NIHSS: National Institutes of Health Stroke Scale.

^a^ Due to the small sample size, the model analysis failed.

**Table S5 Univariate regression Analysis of sICH**

| **sICH** | ***OR*(95%CI) *P*** |
| --- | --- |
| **Gender** |  |
| **male** | 1.0 |
| **female** | 1.31 (0.77, 2.23) 0.3117 |
| **Age** | 1.02 (1.00, 1.04) 0.1154 |
| **NIHSS** | 1.08 (1.05, 1.12) <0.0001 |
| **ASPECTS** | 0.58 (0.40, 0.83) 0.0029 |
| **Pulse** | 1.00 (0.99, 1.01) 0.9815 |
| **Systolic BP** | 1.01 (1.00, 1.02) 0.0347 |
| **Diastolic BP** | 1.02 (1.00, 1.04) 0.0121 |
| **Hypertension（no vs yes）** | 1.39 (0.77, 2.48) 0.2719 |
| **Diabetes mellitus（no vs yes）** | 1.19 (0.62, 2.27) 0.5964 |
| [**Atrial**](javascript:;) [**fibrillation**](javascript:;)**（no vs yes）** | 1.38 (0.82, 2.34) 0.2248 |
| [**Coronary**](javascript:;) [**heart**](javascript:;) [**disease**](javascript:;)**（no vs yes）** | 1.18 (0.49, 2.86) 0.7135 |
| [**Hyperlipemia**](javascript:;)**（no vs yes）** | 0.93 (0.53, 1.64) 0.8052 |
| **Gout（no vs yes）** | 0.72 (0.16, 3.31) 0.6694 |
| **Previous stroke（no vs yes）** | 1.40 (0.75, 2.61) 0.2903 |
| **Smoking（no vs yes）** | 0.69 (0.37, 1.27) 0.2321 |
| **Drinking（no vs yes）** | 0.77 (0.39, 1.49) 0.4317 |
| **TOAST** |  |
| **Large-artery atherosclerosis** | 1.0 |
| **Cardioembolism** | 1.64 (0.94, 2.85) 0.0796 |
| **Othger/undetermined etiology** | 1.23 (0.38, 3.97) 0.7258 |
| **Occlusion site** |  |
| **Intracranial ICA** | 1.0 |
| **MCA** | 0.51 (0.29, 0.91) 0.0227 |
| **Vertebral/basilar artery** | 0.66 (0.28, 1.57) 0.3490 |
| **Infarction site** |  |
| **Left** | 1.0 |
| **Right** | 0.74 (0.44, 1.26) 0.2733 |
| **IV（no vs yes）** | 1.46 (0.86, 2.47) 0.1633 |
| **Threapy** |  |
| **Stent** | 1.0 |
| **Aspiration** | 0.53 (0.19, 1.45) 0.2141 |
| **Stent combined aspiration** | 0.72 (0.32, 1.62) 0.4311 |
| **Other** | 0.54 (0.29, 1.03) 0.0611 |
| **ODT** | 1.00 (1.00, 1.00) 0.2090 |
| **OPT** | 1.00 (0.99, 1.00) 0.0129 |
| **ORT** | 1.00 (1.00, 1.00) 0.0239 |
| **PRT** | 1.00 (1.00, 1.01) 0.4637 |
| **DPT** | 1.00 (0.99, 1.00) 0.0439 |
| **DRT** | 1.00 (0.99, 1.00) 0.1534 |
| [**Recanalization**](javascript:;)**（mTICI 0-2a vs 2b/3）** | 0.57 (0.31, 1.06) 0.0770 |

sICH: Symptomatic intracranial hemorrhage transformation; NIHSS: National Institutes of Health Stroke Scale; ASPECTS: Albert Stroke Project Early CT Score; BP: Blood pressure; TOAST: Trial of Org 10172 in Acute Stroke Treatment; ICA: [Internal](javascript:;) [carotid](javascript:;) artery; MCA: [Middle](javascript:;) [cerebral](javascript:;) [artery](javascript:;); IV: [Intravenous](javascript:;) [thrombolysis](javascript:;); ODT: onset-to-door time; OPT: onset-to-puncture time; ORT: onset-to-recanalization time; PRT: puncture-to-recanalization time; DPT: door-to-puncture time; DRT: door-to-recanalization time.

**Table S6 Univariate regression Analysis of poor outcome**

| **Poor outcome** | ***OR*(95%CI) *P*** |
| --- | --- |
| **Gender** |  |
| **male** | 1.0 |
| **female** | 1.47 (0.93, 2.35) 0.1015 |
| **Age** | 1.03 (1.01, 1.05) 0.0003 |
| **NIHSS** | 1.15 (1.10, 1.21) <0.0001 |
| **ASPECTS** | 0.53 (0.40, 0.72) <0.0001 |
| **Pulse** | 1.01 (1.00, 1.03) 0.0400 |
| **Systolic BP** | 1.01 (1.00, 1.02) 0.0372 |
| **Diastolic BP** | 1.01 (1.00, 1.02) 0.1921 |
| **Hypertension（no vs yes）** | 1.48 (0.93, 2.37) 0.1007 |
| **Diabetes mellitus（no vs yes）** | 1.54 (0.84, 2.82) 0.1589 |
| [**Atrial**](javascript:;) [**fibrillation**](javascript:;)**（no vs yes）** | 1.59 (1.01, 2.53) 0.0472 |
| [**Coronary**](javascript:;) [**heart**](javascript:;) [**disease**](javascript:;)**（no vs yes）** | 1.74 (0.73, 4.16) 0.2146 |
| [**Hyperlipemia**](javascript:;)**（no vs yes）** | 1.01 (0.63, 1.63) 0.9538 |
| **Gout（no vs yes）** | 1.64 (0.44, 6.10) 0.4568 |
| **Previous stroke（no vs yes）** | 1.78 (0.97, 3.28) 0.0633 |
| **Smoking（no vs yes）** | 0.72 (0.44, 1.16) 0.1789 |
| **Drinking（no vs yes）** | 0.70 (0.41, 1.18) 0.1833 |
| **TOAST** |  |
| **Large-artery atherosclerosis** | 1.0 |
| **Cardioembolism** | 1.12 (0.71, 1.79) 0.6253 |
| **Othger/undetermined etiology** | 0.67 (0.26, 1.68) 0.3909 |
| **Occlusion site** |  |
| **Intracranial ICA** | 1.0 |
| **MCA** | 0.44 (0.25, 0.78) 0.0049 |
| **vertebral/basilar artery** | 0.65 (0.29, 1.48) 0.3059 |
| **Infarction site** |  |
| **Left** | 1.0 |
| **Right** | 0.82 (0.53, 1.29) 0.3923 |
| **IV（no vs yes）** | 0.78 (0.50, 1.23) 0.2844 |
| **Threapy** |  |
| **Stent** | 1.0 |
| **Aspiration** | 1.37 (0.58, 3.24) 0.4753 |
| **Stent combined aspiration** | 0.99 (0.49, 2.02) 0.9834 |
| **Other** | 0.59 (0.36, 0.99) 0.0460 |
| **ODT** | 1.00 (1.00, 1.00) 0.5043 |
| **OPT** | 1.00 (1.00, 1.00) 0.4352 |
| **ORT** | 1.00 (1.00, 1.00) 0.6325 |
| **PRT** | 1.00 (1.00, 1.01) 0.3747 |
| **DPT** | 1.00 (1.00, 1.00) 0.4229 |
| **DRT** | 1.00 (1.00, 1.00) 0.7505 |
| [**Recanalization**](javascript:;)**（mTICI 0-2a vs 2b/3）** | 0.24 (0.11, 0.53) 0.0003 |

NIHSS: National Institutes of Health Stroke Scale; ASPECTS: Albert Stroke Project Early CT Score; BP: Blood pressure; TOAST: Trial of Org 10172 in Acute Stroke Treatment; ICA: [Internal](javascript:;) [carotid](javascript:;) artery; MCA: [Middle](javascript:;) [cerebral](javascript:;) [artery](javascript:;); IV: [Intravenous](javascript:;) [thrombolysis](javascript:;); ODT: onset-to-door time; OPT: onset-to-puncture time; ORT: onset-to-recanalization time; PRT: puncture-to-recanalization time; DPT: door-to-puncture time; DRT: door-to-recanalization time.

**Table S7 Univariate regression Analysis of mortality**

| **Mortality** | ***OR(*95%CI) *P*** |
| --- | --- |
| **Gender** |  |
| **male** | 1.0 |
| **female** | 1.32 (0.82, 2.12) 0.2582 |
| **Age** | 1.04 (1.02, 1.06) 0.0004 |
| **NIHSS** | 1.16 (1.11, 1.20) <0.0001 |
| **ASPECTS** | 0.45 (0.32, 0.63) <0.0001 |
| **Pulse** | 1.01 (1.00, 1.02) 0.1628 |
| **Systolic BP** | 1.02 (1.01, 1.03) 0.0028 |
| **Diastolic BP** | 1.02 (1.00, 1.03) 0.0299 |
| **Hypertension（no vs yes）** | 2.20 (1.26, 3.84) 0.0053 |
| **Diabetes mellitus（no vs yes）** | 1.52 (0.86, 2.70) 0.1477 |
| [**Atrial**](javascript:;) [**fibrillation**](javascript:;)**（no vs yes）** | 1.17 (0.73, 1.87) 0.5245 |
| [**Coronary**](javascript:;) [**heart**](javascript:;) [**disease**](javascript:;)**（no vs yes）** | 1.31 (0.59, 2.88) 0.5102 |
| [**Hyperlipemia**](javascript:;)**（no vs yes）** | 0.82 (0.49, 1.36) 0.4373 |
| **Gout（no vs yes）** | 1.20 (0.36, 3.98) 0.7698 |
| **Previous stroke（no vs yes）** | 1.70 (0.97, 2.99) 0.0622 |
| **Smoking（no vs yes）** | 0.73 (0.43, 1.26) 0.2626 |
| **Drinking（no vs yes）** | 0.84 (0.47, 1.51) 0.5700 |
| **TOAST** |  |
| **Large-artery atherosclerosis** | 1.0 |
| **Cardioembolism** | 1.26 (0.78, 2.06) 0.3462 |
| **Othger/undetermined etiology** | 0.94 (0.32, 2.73) 0.9057 |
| **Occlusion site** |  |
| **Intracranial ICA** | 1.0 |
| **MCA** | 0.33 (0.19, 0.58) <0.0001 |
| **vertebral/basilar artery** | 1.58 (0.77, 3.26) 0.2131 |
| **Infarction site** |  |
| **Left** | 1.0 |
| **Right** | 0.68 (0.42, 1.10) 0.1147 |
| **IV（no vs yes）** | 0.65 (0.40, 1.08) 0.0942 |
| **Threapy** |  |
| **Stent** | 1.0 |
| **Aspiration** | 1.33 (0.61, 2.91) 0.4688 |
| **Stent combined aspiration** | 0.58 (0.26, 1.29) 0.1824 |
| **Other** | 0.84 (0.48, 1.45) 0.5264 |
| **ODT** | 1.00 (1.00, 1.00) 0.2582 |
| **OPT** | 1.00 (1.00, 1.00) 0.0230 |
| **ORT** | 1.00 (1.00, 1.00) 0.0271 |
| **PRT** | 1.00 (0.99, 1.01) 0.9232 |
| **DPT** | 0.99 (0.99, 1.00) 0.0115 |
| **DRT** | 1.00 (0.99, 1.00) 0.0246 |
| [**Recanalization**](javascript:;)**（mTICI 0-2a vs 2b/3）** | 0.28 (0.16, 0.50) <0.0001 |

NIHSS: National Institutes of Health Stroke Scale; ASPECTS: Albert Stroke Project Early CT Score; BP: Blood pressure; TOAST: Trial of Org 10172 in Acute Stroke Treatment; ICA: [Internal](javascript:;) [carotid](javascript:;) artery; MCA: [Middle](javascript:;) [cerebral](javascript:;) [artery](javascript:;); IV: [Intravenous](javascript:;) [thrombolysis](javascript:;); ODT: onset-to-door time; OPT: onset-to-puncture time; ORT: onset-to-recanalization time; PRT: puncture-to-recanalization time; DPT: door-to-puncture time; DRT: door-to-recanalization time.

**Table S8 Univariate regression Analysis of cost**

| **Cost** | ***β*(95%CI) *P*** |
| --- | --- |
| **Gender** |  |
| **male** | 0 |
| **female** | 1315.83 (-11018.09, 13649.76) 0.8345 |
| **Age** | 46.60 (-403.44, 496.65) 0.8393 |
| **NIHSS** | 597.39 (-173.94, 1368.71) 0.1299 |
| **ASPECTS** | -4402.53 (-11888.69, 3083.64) 0.2498 |
| **Pulse** | 6.33 (-323.27, 335.93) 0.9700 |
| **Systolic BP** | -32.03 (-294.79, 230.74) 0.8113 |
| **Diastolic BP** | -86.57 (-460.82, 287.67) 0.6505 |
| **Hypertension（no vs yes）** | -3781.40 (-16681.02, 9118.22) 0.5660 |
| **Diabetes mellitus（no vs yes）** | 1482.34 (-13884.53, 16849.22) 0.8501 |
| [**Atrial**](javascript:;) [**fibrillation**](javascript:;)**（no vs yes）** | 841.18 (-11365.67, 13048.03) 0.8926 |
| [**Coronary**](javascript:;) [**heart**](javascript:;) [**disease**](javascript:;)**（no vs yes）** | -9344.63 (-30597.19, 11907.93) 0.3894 |
| [**Hyperlipemia**](javascript:;)**（no vs yes）** | 2704.37 (-10198.25, 15606.99) 0.6815 |
| **Gout（no vs yes）** | -14369.90 (-46295.72, 17555.92) 0.3783 |
| **Previous stroke（no vs yes）** | -4870.88 (-20059.53, 10317.77) 0.5301 |
| **Smoking（no vs yes）** | -2872.67 (-16255.85, 10510.51) 0.6742 |
| **Drinking（no vs yes）** | -4538.54 (-19126.93, 10049.85) 0.5424 |
| **TOAST** |  |
| **Large-artery atherosclerosis** | 0 |
| **Cardioembolism** | 1614.66 (-10902.60, 14131.92) 0.8005 |
| **Othger/undetermined etiology** | 4322.45 (-21993.17, 30638.07) 0.7477 |
| **Occlusion site** |  |
| **Intracranial ICA** | 0 |
| **MCA** | 5593.31 (-8310.85, 19497.47) 0.4310 |
| **vertebral/basilar artery** | 30520.11 (10065.24, 50974.98) 0.0037 |
| **Infarction site** |  |
| **Left** | 0 |
| **Right** | -7007.15 (-19132.03, 5117.73) 0.2581 |
| **IV（no vs yes）** | 6336.94 (-6008.90, 18682.77) 0.3151 |
| **Threapy** |  |
| **Stent** | 0 |
| **Aspiration** | 18835.08 (-1768.24, 39438.40) 0.0740 |
| **Stent combined aspiration** | 37782.99 (19687.39, 55878.58) <0.0001 |
| **Other** | -9677.69 (-23322.36, 3966.99) 0.1654 |
| **ODT** | -16.67 (-57.33, 23.98) 0.4221 |
| **OPT** | -15.22 (-55.23, 24.78) 0.4562 |
| **ORT** | -2.68 (-42.12, 36.77) 0.8943 |
| **PRT** | 200.76 (37.85, 363.67) 0.0162 |
| **DPT** | -17.50 (-99.48, 64.48) 0.6759 |
| **DRT** | 26.69 (-46.93, 100.31) 0.4778 |
| [**Recanalization**](javascript:;)**（mTICI 0-2a vs 2b/3）** | -5939.88 (-21568.14, 9688.37) 0.4568 |

NIHSS: National Institutes of Health Stroke Scale; ASPECTS: Albert Stroke Project Early CT Score; BP: Blood pressure; TOAST: Trial of Org 10172 in Acute Stroke Treatment; ICA: [Internal](javascript:;) [carotid](javascript:;) artery; MCA: [Middle](javascript:;) [cerebral](javascript:;) [artery](javascript:;); IV: [Intravenous](javascript:;) [thrombolysis](javascript:;); ODT: onset-to-door time; OPT: onset-to-puncture time; ORT: onset-to-recanalization time; PRT: puncture-to-recanalization time; DPT: door-to-puncture time; DRT: door-to-recanalization time.

**Table S9 Collinear screening**

| **Variable** | **VIF** |
| --- | --- |
| **Gender** | 1.7 |
| **Age** | 1.9 |
| **Pulse** | 1.5 |
| **Systolic BP** | 2 |
| **Diastolic BP** | 2 |
| **ASPECTS** | 1.2 |
| **NIHSS** | 1.3 |
| **Hypertension** | 1.3 |
| **Diabetes mellitus** | 1.1 |
| [**Atrial**](javascript:;) [**fibrillation**](javascript:;) | 2.1 |
| [**Coronary**](javascript:;) [**heart**](javascript:;) [**disease**](javascript:;) | 1.1 |
| [**Hyperlipemia**](javascript:;) | 1.1 |
| **Gout** | 1.1 |
| **Previous stroke** | 1.1 |
| **Smoking** | 2 |
| **Drinking** | 1.8 |
| **TOAST** | 1.8 |
| **IV** | 1.1 |
| **Occlusion site** | 1.2 |
| **Infarction site** | 1.1 |
| **Threapy** | 1.2 |
| **ODT** | NA |
| **OPT** | NA |
| **DPT** | NA |
| **ORT** | 1.3 |
| **PRT** | 1.6 |
| **DRT** | 1.5 |
| [**Recanalization**](javascript:;) | 1.1 |

VIF: [variance](javascript:;) [inflation](javascript:;) [factor](javascript:;) ; BP: Blood pressure; ASPECTS: Albert Stroke Project Early CT Score; NIHSS: National Institutes of Health Stroke Scale; TOAST: Trial of Org 10172 in Acute Stroke Treatment; IV: [Intravenous](javascript:;) [thrombolysis](javascript:;);

ODT: onset-to-door time; OPT: onset-to-puncture time; DPT: door-to-puncture time; ORT: onset-to-recanalization time; PRT: puncture-to-recanalization time; DRT: door-to-recanalization time.

If the variance [inflation](javascript:;) factor is greater than or equal to 5, collinearity is considered to exist, and these covariables are eliminated in the final model; excluded variables: ODT, OPT, DPT.

**Table S10 The relationship between covariates and the sICH（n=349）**

| **Covariates** | **exp(beta)** | **95%CI** | ***P* value** |
| --- | --- | --- | --- |
| **Gender** | 1.3134 | 0.7745-2.2273 | 0.3117 |
| **Age** | 1.0166 | 0.9960-1.0378 | 0.1154 |
| **Occlusion site** |  |  |  |
| **Intracranial ICA** | Ref | Ref | Ref |
| **MCA** | 0.5096 | 0.2853-0.9102 | 0.0227 |
| **Vertebral/basilar artery** | 0.6626 | 0.2801-1.5678 | 0.3490 |
| **Occlusion site** | 0.7424 | 0.4358-1.2649 | 0.2733 |
| **Hypertension** | 1.3859 | 0.7743-2.4804 | 0.2719 |
| **Diabetes mellitus** | 1.1906 | 0.6243-2.2705 | 0.5964 |
| [**Atrial**](javascript:;) [**fibrillation**](javascript:;) | 1.3846 | 0.8187-2.3416 | 0.2248 |
| [**Coronary**](javascript:;) [**heart**](javascript:;) [**disease**](javascript:;) | 1.1806 | 0.4868-2.8632 | 0.7135 |
| [**Hyperlipemia**](javascript:;) | 0.9316 | 0.5306-1.6358 | 0.8052 |
| **Gout** | 0.7166 | 0.1552-3.3092 | 0.6694 |
| **Previous stroke** | 1.4002 | 0.7503-2.6131 | 0.2903 |
| **Smoking** | 0.6878 | 0.3723-1.2706 | 0.2321 |
| **Drinking** | 0.7662 | 0.3944-1.4882 | 0.4317 |
| **Systolic BP** | 1.0120 | 1.0009-1.0233 | 0.0347 |
| **ASPECTS** | 0.5753 | 0.4000-0.8276 | 0.0029 |
| **Diastolic BP** | 1.0205 | 1.0045-1.0368 | 0.0121 |
| **Pulse** | 1.0002 | 0.9860-1.0145 | 0.9815 |
| **TOAST** |  |  |  |
| **Large-artery atherosclerosis** | Ref | Ref | Ref |
| **Cardioembolism** | 1.6400 | 0.9432-2.8516 | 0.0796 |
| **Othger/undetermined etiology** | 1.2329 | 0.3826-3.9733 | 0.7258 |
| **IV** | 1.4556 | 0.8586-2.4679 | 0.1633 |
| **NIHSS** | 1.0838 | 1.0502-1.1184 | <0.0001 |
| **Threapy** |  |  |  |
| **Stent** | Ref | Ref | Ref |
| **Aspiration** | 0.5259 | 0.1908-1.4497 | 0.2141 |
| **Stent combined aspiration** | 0.7224 | 0.3215-1.6232 | 0.4311 |
| **Other** | 0.5422 | 0.2858-1.0289 | 0.0611 |
| **ORT** | 0.9974 | 0.9952-0.9997 | 0.0239 |
| **PRT** | 1.0026 | 0.9957-1.0095 | 0.4637 |
| **DRT** | 0.9974 | 0.9939-1.0010 | 0.1534 |
| [**Recanalization**](javascript:;) | 0.5703 | 0.3061-1.0627 | 0.0770 |

Ref: Reference; sICH: Symptomatic intracranial hemorrhage transformation; ICA: [Internal](javascript:;) [carotid](javascript:;) artery; MCA: [Middle](javascript:;) [cerebral](javascript:;) [artery](javascript:;); BP: Blood pressure; ASPECTS: Albert Stroke Project Early CT Score; TOAST: Trial of Org 10172 in Acute Stroke Treatment; IV: [Intravenous](javascript:;) [thrombolysis](javascript:;); NIHSS: National Institutes of Health Stroke Scale; ORT: onset-to-recanalization time; PRT: puncture-to-recanalization time; DRT: door-to-recanalization time.

**Table S11 The relationship between covariates and poor outcome（n=349）**

| **Covariates** | **exp(beta)** | **95%CI** | ***P* value** |
| --- | --- | --- | --- |
| **Gender** | 1.4748 | 0.9264-2.3481 | 0.1015 |
| **Age** | 1.0318 | 1.0145-1.0494 | 0.0003 |
| **Occlusion site** |  |  |  |
| **Intracranial ICA** | Ref | Ref | Ref |
| **MCA** | 0.4431 | 0.2513-0.7811 | 0.0049 |
| **Vertebral/basilar artery** | 0.6533 | 0.2892-1.4759 | 0.3059 |
| **Occlusion site** | 0.8222 | 0.5251-1.2875 | 0.3923 |
| **Hypertension** | 1.4816 | 0.9266-2.3692 | 0.1007 |
| **Diabetes mellitus** | 1.5421 | 0.8441-2.8174 | 0.1589 |
| [**Atrial**](javascript:;) [**fibrillation**](javascript:;) | 1.5938 | 1.0058-2.5256 | 0.0472 |
| [**Coronary**](javascript:;) [**heart**](javascript:;) [**disease**](javascript:;) | 1.7387 | 0.7259-4.1645 | 0.2146 |
| [**Hyperlipemia**](javascript:;) | 1.0142 | 0.6292-1.6348 | 0.9538 |
| **Gout** | 1.6444 | 0.4437-6.0952 | 0.4568 |
| **Previous stroke** | 1.7837 | 0.9685-3.2848 | 0.0633 |
| **Smoking** | 0.7168 | 0.4410-1.1648 | 0.1789 |
| **Drinking** | 0.7000 | 0.4140-1.1837 | 0.1833 |
| **Systolic BP** | 1.0109 | 1.0006-1.0212 | 0.0372 |
| **ASPECTS** | 0.5347 | 0.3995-0.7157 | <0.0001 |
| **Diastolic BP** | 1.0095 | 0.9953-1.0239 | 0.1921 |
| **Pulse** | 1.0138 | 1.0006-1.0271 | 0.0400 |
| **TOAST** |  |  |  |
| **Large-artery atherosclerosis** | Ref | Ref | Ref |
| **Cardioembolism** | 1.1226 | 0.7057-1.7860 | 0.6253 |
| **Othger/undetermined etiology** | 0.6667 | 0.2640-1.6832 | 0.3909 |
| **IV** | 0.7803 | 0.4954-1.2289 | 0.2844 |
| **NIHSS** | 1.1518 | 1.0967-1.2096 | <0.0001 |
| **Threapy** |  |  |  |
| **Stent** | Ref | Ref | Ref |
| **Aspiration** | 1.3684 | 0.5784-3.2377 | 0.4753 |
| **Stent combined aspiration** | 0.9925 | 0.4878-2.0193 | 0.9834 |
| **Other** | 0.5933 | 0.3553-0.9908 | 0.0460 |
| **ORT** | 0.9997 | 0.9982-1.0011 | 0.6325 |
| **PRT** | 1.0028 | 0.9966-1.0091 | 0.3747 |
| **DRT** | 0.9996 | 0.9969-1.0023 | 0.7505 |
| [**Recanalization**](javascript:;) | 0.2412 | 0.1107-0.5256 | 0.0003 |

Ref: Reference; ICA: [Internal](javascript:;) [carotid](javascript:;) artery; MCA: [Middle](javascript:;) [cerebral](javascript:;) [artery](javascript:;); BP: Blood pressure; ASPECTS: Albert Stroke Project Early CT Score; TOAST: Trial of Org 10172 in Acute Stroke Treatment; IV: [Intravenous](javascript:;) [thrombolysis](javascript:;); NIHSS: National Institutes of Health Stroke Scale; ORT: onset-to-recanalization time; PRT: puncture-to-recanalization time; DRT: door-to-recanalization time.

**Table S12 The relationship between covariates and mortality（n=349）**

| **Covariates** | **exp(beta)** | **95%CI** | ***P* value** |
| --- | --- | --- | --- |
| **Gender** | 1.3163 | 0.8174-2.1198 | 0.2582 |
| **Age** | 1.0363 | 1.0159-1.0571 | 0.0004 |
| **Occlusion site** |  |  |  |
| **Intracranial ICA** | Ref | Ref | Ref |
| **MCA** | 0.3348 | 0.1936-0.5788 | 0.0001 |
| **Vertebral/basilar artery** | 1.5833 | 0.7681-3.2640 | 0.2131 |
| **Occlusion site** | 0.6794 | 0.4203-1.0982 | 0.1147 |
| **Hypertension** | 2.2031 | 1.2646-3.8382 | 0.0053 |
| **Diabetes mellitus** | 1.5246 | 0.8614-2.6985 | 0.1477 |
| [**Atrial**](javascript:;) [**fibrillation**](javascript:;) | 1.1663 | 0.7262-1.8732 | 0.5245 |
| [**Coronary**](javascript:;) [**heart**](javascript:;) [**disease**](javascript:;) | 1.3053 | 0.5907-2.8847 | 0.5102 |
| [**Hyperlipemia**](javascript:;) | 0.8164 | 0.4893-1.3621 | 0.4373 |
| **Gout** | 1.1966 | 0.3596-3.9813 | 0.7698 |
| **Previous stroke** | 1.7045 | 0.9732-2.9856 | 0.0622 |
| **Smoking** | 0.7343 | 0.4278-1.2605 | 0.2626 |
| **Drinking** | 0.8448 | 0.4721-1.5117 | 0.5700 |
| **Systolic BP** | 1.0158 | 1.0054-1.0263 | 0.0028 |
| **ASPECTS** | 0.4470 | 0.3160-0.6323 | <0.0001 |
| **Diastolic BP** | 1.0162 | 1.0016-1.0310 | 0.0299 |
| **Pulse** | 1.0090 | 0.9964-1.0217 | 0.1628 |
| **TOAST** |  |  |  |
| **Large-artery atherosclerosis** | Ref | Ref | Ref |
| **Cardioembolism** | 1.2645 | 0.7760-2.0604 | 0.3462 |
| **Othger/undetermined etiology** | 0.9375 | 0.3223-2.7266 | 0.9057 |
| **IV** | 0.6548 | 0.3988-1.0751 | 0.0942 |
| **NIHSS** | 1.1575 | 1.1130-1.2039 | <0.0001 |
| **Threapy** |  |  |  |
| **Stent** | Ref | Ref | Ref |
| **Aspiration** | 1.3346 | 0.6112-2.9142 | 0.4688 |
| **Stent combined aspiration** | 0.5795 | 0.2599-1.2922 | 0.1824 |
| **Other** | 0.8363 | 0.4809-1.4541 | 0.5264 |
| **ORT** | 0.9979 | 0.9960-0.9998 | 0.0271 |
| **PRT** | 1.0003 | 0.9940-1.0067 | 0.9232 |
| **DRT** | 0.9963 | 0.9930-0.9995 | 0.0246 |
| [**Recanalization**](javascript:;) | 0.2838 | 0.1614-0.4989 | <0.0001 |

Ref: Reference; ICA: [Internal](javascript:;) [carotid](javascript:;) artery; MCA: [Middle](javascript:;) [cerebral](javascript:;) [artery](javascript:;); BP: Blood pressure; ASPECTS: Albert Stroke Project Early CT Score; TOAST: Trial of Org 10172 in Acute Stroke Treatment; IV: [Intravenous](javascript:;) [thrombolysis](javascript:;); NIHSS: National Institutes of Health Stroke Scale; ORT: onset-to-recanalization time; PRT: puncture-to-recanalization time; DRT: door-to-recanalization time.

**Table S13 The relationship between covariates and cost（n=349）**

| **Covariates** | beta | 95%CI | | *P* value |
| --- | --- | --- | --- | --- |
| **Gender** | 1315.8340 | -11018.0901-13649.7581 | | 0.8345 |
| **Age** | 46.6030 | -403.4400-496.6459 | | 0.8393 |
| **Occlusion site** |  |  | |  |
| **Intracranial ICA** | Ref | Ref | | Ref |
| **MCA** | 5593.3095 | -8310.8515-19497.4705 | | 0.4310 |
| **Vertebral/basilar artery** | 30520.1092 | 10065.2383-50974.9802 | | 0.0037 |
| **Occlusion site** | -7007.1516 | -19132.0340-5117.7309 | | 0.2581 |
| **Hypertension** | -3781.4000 | -16681.0230-9118.2230 | | 0.5660 |
| **Diabetes mellitus** | 1482.3430 | -13884.5310-16849.2171 | | 0.8501 |
| [**Atrial**](javascript:;) [**fibrillation**](javascript:;) | 841.1789 | -11365.6690-13048.0268 | | 0.8926 |
| [**Coronary**](javascript:;) [**heart**](javascript:;) [**disease**](javascript:;) | -9344.6304 | -30597.1866-11907.9259 | | 0.3894 |
| [**Hyperlipemia**](javascript:;) | 2704.3722 | -10198.2480-15606.9924 | | 0.6815 |
| **Gout** | -14369.8997 | -46295.7190-17555.9196 | | 0.3783 |
| **Previous stroke** | -4870.8834 | -20059.5342-10317.7674 | | 0.5301 |
| **Smoking** | -2872.6696 | -16255.8536-10510.5144 | | 0.6742 |
| **Drinking** | -4538.5406 | -19126.9347-10049.8536 | | 0.5424 |
| **Systolic BP** | -32.0253 | -294.7923-230.7417 | | 0.8113 |
| **ASPECTS** | -4402.5260 | -11888.6948-3083.6429 | | 0.2498 |
| **Diastolic BP** | -86.5727 | -460.8194-287.6740 | | 0.6505 |
| **Pulse** | 6.3298 | -323.2734-335.9331 | | 0.9700 |
| **TOAST** |  |  |  |  |
| **Large-artery atherosclerosis** | Ref | Ref | | Ref |
| **Cardioembolism** | 1614.6635 | -10902.5966-14131.9237 | | 0.8005 |
| **Othger/undetermined etiology** | 4322.4515 | -21993.1661-30638.0691 | | 0.7477 |
| **IV** | 6336.9370 | -6008.8951-18682.7690 | | 0.3151 |
| **NIHSS** | 597.3887 | -173.9376-1368.7149 | | 0.1299 |
| **Threapy** |  |  | |  |
| **Stent** | Ref | Ref | | Ref |
| **Aspiration** | 18835.0788 | -1768.2449-39438.4025 | | 0.0740 |
| **Stent combined aspiration** | 37782.9856 | 19687.3883-55878.5828 | | 0.0001 |
| **Other** | -9677.6865 | -23322.3607-3966.9878 | | 0.1654 |
| **ORT** | -2.6755 | -42.1217-36.7707 | | 0.8943 |
| **PRT** | 200.7572 | 37.8461-363.6683 | | 0.0162 |
| **DRT** | 26.6903 | -46.9307-100.3113 | | 0.4778 |
| [**Recanalization**](javascript:;) | -5939.8834 | -21568.1368-9688.3699 | | 0.4568 |

Ref: Reference; ICA: [Internal](javascript:;) [carotid](javascript:;) artery; MCA: [Middle](javascript:;) [cerebral](javascript:;) [artery](javascript:;); BP: Blood pressure; ASPECTS: Albert Stroke Project Early CT Score; TOAST: Trial of Org 10172 in Acute Stroke Treatment; IV: [Intravenous](javascript:;) [thrombolysis](javascript:;); NIHSS: National Institutes of Health Stroke Scale; ORT: onset-to-recanalization time; PRT: puncture-to-recanalization time; DRT: door-to-recanalization time.

**Table S14 The adjusting effect of potential mixed factors on the estimated value of sICH**

| **+/- Covariates** |  | **Basic model** |  | **Complete model** |  |  |
| --- | --- | --- | --- | --- | --- | --- |
|  | **DD** | **MS** | **DS** | **MS** | **DS** | **Pick** |
| **IRC** | Ref | -0.2471 | -0.2138 | -0.5949 | -0.6391 |  |
| **Gender** | Ref | -0.2723 * | -0.2199 * | -0.6039 | -0.6440 | Yes |
| **Age** | Ref | -0.2440 | -0.1851 | -0.6002 * | -0.6622 * | Yes |
| **Occlusion site** | Ref | -0.1276 * | -0.1959 * | -0.6018 | -0.5152 | Yes |
| **Infarction site** | Ref | -0.2206 * | -0.1942 * | -0.5975 | -0.6480 | Yes |
| **Hypertension** | Ref | -0.2629 | -0.2455 | -0.5962 * | -0.6451 * | Yes |
| **Diabetes mellitus** | Ref | -0.2293 | -0.2072 | -0.6114 | -0.6457 |  |
| [**Atrial**](javascript:;) [**fibrillation**](javascript:;) | Ref | -0.2685 | -0.2142 | -0.5462 | -0.6410 |  |
| [**Coronary**](javascript:;) [**heart**](javascript:;) [**disease**](javascript:;) | Ref | -0.2469 | -0.2199 | -0.5785 | -0.6651 |  |
| [**Hyperlipemia**](javascript:;) | Ref | -0.2576 | -0.2145 | -0.6119 | -0.6378 |  |
| **Gout** | Ref | -0.2589 | -0.2153 | -0.5546 | -0.6317 |  |
| **Previous stroke** | Ref | -0.2218 * | -0.1983 * | -0.6009 | -0.6444 | Yes |
| **Smoking** | Ref | -0.2296 | -0.2088 | -0.6083 | -0.6409 |  |
| **Drinking** | Ref | -0.2312 | -0.2086 | -0.6005 | -0.6409 |  |
| **Systolic BP** | Ref | -0.2455 | -0.1998 | -0.5807 | -0.6362 |  |
| **ASPECTS** | Ref | -0.5456 * | -0.4600 * | -0.2634 * | -0.3955 * | Yes |
| **Diastolic BP** | Ref | -0.2196 * | -0.2032 * | -0.5928 | -0.6113 | Yes |
| **Pulse** | Ref | -0.2521 | -0.2177 | -0.6096 | -0.6248 |  |
| **TOAST** | Ref | -0.3180 * | -0.2302 * | -0.4906 | -0.6408 | Yes |
| **IV** | Ref | -0.1647 * | -0.1236 * | -0.6480 * | -0.7260 * | Yes |
| **NIHSS** | Ref | -0.3288 * | -0.3807 * | -0.5173 * | -0.4618 * | Yes |
| **Threapy** | Ref | -0.3034 * | -0.2642 * | -0.5702 * | -0.6132 * | Yes |
| **ORT** | Ref | -0.1793 * | -0.1515 * | -0.6578 * | -0.6896 * | Yes |
| **PRT** | Ref | -0.1924 * | -0.1547 * | -0.5817 * | -0.6223 * | Yes |
| **DRT** | Ref | -0.4561 * | -0.4023 * | -0.4677 * | -0.5224 * | Yes |
| [**Recanalization**](javascript:;) | Ref | -0.2169 * | -0.2341 * | -0.6065 | -0.6198 | Yes |

sICH: Symptomatic intracranial hemorrhage transformation; DD: Drive the Doctor; MS: Mothership; DS: Drip and Ship; Ref: Reference; IRC: Initial regression coefficient; BP: Blood pressure; ASPECTS: Albert Stroke Project Early CT Score; TOAST: Trial of Org 10172 in Acute Stroke Treatment; IV: [Intravenous](javascript:;) [thrombolysis](javascript:;); NIHSS: National Institutes of Health Stroke Scale; ORT: onset-to-recanalization time; PRT: puncture-to-recanalization time; DRT: door-to-recanalization time.

* indicates a change of more than 10% compared with the initial regression coefficient.

**Table S15 The adjusting effect of potential mixed factors on the estimated value of poor outcome**

| **+/- Covariates** |  | **Basic model** |  | **Complete**  **model** |  |  |
| --- | --- | --- | --- | --- | --- | --- |
|  | **DD** | **MS** | **DS** | **MS** | **DS** | **Pick** |
| **IRC** | Ref | -0.1234 | 0.0477 | -0.4677 | -0.6221 |  |
| **Gender** | Ref | -0.1582 * | 0.0407 * | -0.4655 * | -0.6221 * | Yes |
| **Age** | Ref | -0.1252 | 0.1263 | -0.4946 * | -0.6546 * | Yes |
| **Occlusion site** | Ref | 0.0029 * | 0.0718 * | -0.5417 * | -0.5865 * | Yes |
| **Infarction site** | Ref | -0.1049 * | 0.0618 * | -0.4647 * | -0.6201 * | Yes |
| **Hypertension** | Ref | -0.1421 * | 0.0110 * | -0.4754 * | -0.6350 * | Yes |
| **Diabetes mellitus** | Ref | -0.0788 * | 0.0651 * | -0.5044 * | -0.6362 * | Yes |
| [**Atrial**](javascript:;) [**fibrillation**](javascript:;) | Ref | -0.1537 * | 0.0491 * | -0.5061 | -0.6321 | Yes |
| [**Coronary**](javascript:;) [**heart**](javascript:;) [**disease**](javascript:;) | Ref | -0.1230 | 0.0317 | -0.4691 * | -0.6232 * | Yes |
| [**Hyperlipemia**](javascript:;) | Ref | -0.1236 | 0.0477 | -0.5049 | -0.6100 |  |
| **Gout** | Ref | -0.1077 * | 0.0495 * | -0.4736 | -0.6230 | Yes |
| **Previous stroke** | Ref | -0.0833 * | 0.0740 * | -0.4698 * | -0.6274 * | Yes |
| **Smoking** | Ref | -0.1063 * | 0.0536 * | -0.4581 * | -0.6201 * | Yes |
| **Drinking** | Ref | -0.1001 * | 0.0562 * | -0.4709 * | -0.6275 * | Yes |
| **Systolic BP** | Ref | -0.1044 * | 0.0645 * | -0.4633 * | -0.6226 * | Yes |
| **ASPECTS** | Ref | -0.4598 * | -0.2186 * | -0.2189 * | -0.4116 * | Yes |
| **Diastolic BP** | Ref | -0.1029 * | 0.0565 * | -0.4811 * | -0.6297 * | Yes |
| **Pulse** | Ref | -0.2078 * | -0.0145 * | -0.4077 * | -0.5774 * | Yes |
| **TOAST** | Ref | -0.1366 * | 0.0420 * | -0.5310 * | -0.6137 * | Yes |
| **IV** | Ref | -0.1858 * | -0.0198 * | -0.4016 * | -0.5026 * | Yes |
| **NIHSS** | Ref | -0.3300 * | -0.1789 * | -0.2959 * | -0.3040 * | Yes |
| **Threapy** | Ref | -0.2521 * | -0.0679 * | -0.3981 * | -0.5065 * | Yes |
| **ORT** | Ref | -0.1120 | 0.0589 | -0.3578 * | -0.5082 * | Yes |
| **PRT** | Ref | -0.0352 * | 0.1440 * | -0.4491 * | -0.5938 * | Yes |
| **DRT** | Ref | -0.1594 * | 0.0160 * | -0.5013 * | -0.6429 * | Yes |
| [**Recanalization**](javascript:;) | Ref | -0.0688 * | 0.0156 * | -0.4534 * | -0.4967 * | Yes |

DD: Drive the Doctor; MS: Mothership; DS: Drip and Ship; Ref: Reference; IRC: Initial regression coefficient; BP: Blood pressure; ASPECTS: Albert Stroke Project Early CT Score; TOAST: Trial of Org 10172 in Acute Stroke Treatment; IV: [Intravenous](javascript:;) [thrombolysis](javascript:;); NIHSS: National Institutes of Health Stroke Scale; ORT: onset-to-recanalization time; PRT: puncture-to-recanalization time; DRT: door-to-recanalization time.

* indicates a change of more than 10% compared with the initial regression coefficient.

**Table S16 The adjusting effect of potential mixed factors on the estimated value of mortality**

| **+/- Covariates** |  | **Basic model** |  | **Complete model** |  |  |
| --- | --- | --- | --- | --- | --- | --- |
|  | **DD** | **MS** | **DS** | **MS** | **DS** | **Pick** |
| **IRC** | Ref | 0.0330 | 0.2922 | -0.5555 | -0.3830 |  |
| **Gender** | Ref | 0.0087 * | 0.2879 * | -0.5561 | -0.3852 | Yes |
| **Age** | Ref | 0.0484 * | 0.3652 * | -0.5519 * | -0.4756 * | Yes |
| **Occlusion site** | Ref | 0.2246 * | 0.3425 * | -0.5917 * | -0.2700 * | Yes |
| **Infarction site** | Ref | 0.0713 * | 0.3227 * | -0.5670 * | -0.4101 * | Yes |
| **Hypertension** | Ref | 0.0013 * | 0.2311 * | -0.5549 * | -0.3741 * | Yes |
| **Diabetes mellitus** | Ref | 0.0838 * | 0.3130 * | -0.6120 | -0.4079 | Yes |
| [**Atrial**](javascript:;) [**fibrillation**](javascript:;) | Ref | 0.0231 * | 0.2929 * | -0.5278 | -0.4001 | Yes |
| [**Coronary**](javascript:;) [**heart**](javascript:;) [**disease**](javascript:;) | Ref | 0.0335 | 0.2843 | -0.5563 | -0.4008 |  |
| [**Hyperlipemia**](javascript:;) | Ref | 0.0058 * | 0.2911 * | -0.5887 | -0.3664 | Yes |
| **Gout** | Ref | 0.0390 * | 0.2929 * | -0.5671 | -0.3844 | Yes |
| **Previous stroke** | Ref | 0.0783 * | 0.3232 * | -0.5636 * | -0.3975 * | Yes |
| **Smoking** | Ref | 0.0488 * | 0.2982 * | -0.5640 | -0.3886 | Yes |
| **Drinking** | Ref | 0.0438 * | 0.2961 * | -0.5539 | -0.3818 | Yes |
| **Systolic BP** | Ref | 0.0387 * | 0.3210 * | -0.5185 | -0.3669 | Yes |
| **ASPECTS** | Ref | -0.3501 * | -0.0046 * | -0.1475 * | -0.0946 * | Yes |
| **Diastolic BP** | Ref | 0.0621 * | 0.3083 * | -0.5933 | -0.3964 | Yes |
| **Pulse** | Ref | -0.0190 * | 0.2527 * | -0.5700 * | -0.3873 * | Yes |
| **TOAST** | Ref | 0.0011 * | 0.2856 * | -0.4515 | -0.4200 | Yes |
| **IV** | Ref | -0.0611 * | 0.1915 * | -0.3923 * | -0.1825 * | Yes |
| **NIHSS** | Ref | -0.0568 * | 0.1479 * | -0.3505 * | -0.0947 * | Yes |
| **Threapy** | Ref | -0.0190 * | 0.2684 * | -0.5658 | -0.4106 | Yes |
| **ORT** | Ref | 0.0973 * | 0.3565 * | -0.5901 * | -0.4054 * | Yes |
| **PRT** | Ref | 0.0729 * | 0.3354 * | -0.5733 * | -0.4082 * | Yes |
| **DRT** | Ref | -0.2012 * | 0.0846 * | -0.4041 * | -0.2589 * | Yes |
| [**Recanalization**](javascript:;) | Ref | 0.1130 * | 0.2675 * | -0.5861 | -0.2788 | Yes |

DD: Drive the Doctor; MS: Mothership; DS: Drip and Ship; Ref: Reference; IRC: Initial regression coefficient; BP: Blood pressure; ASPECTS: Albert Stroke Project Early CT Score; TOAST: Trial of Org 10172 in Acute Stroke Treatment; IV: [Intravenous](javascript:;) [thrombolysis](javascript:;); NIHSS: National Institutes of Health Stroke Scale; ORT: onset-to-recanalization time; PRT: puncture-to-recanalization time; DRT: door-to-recanalization time.

* indicates a change of more than 10% compared with the initial regression coefficient.

**Table S17 The adjusting effect of potential mixed factors on the estimated value of cost**

| +/- **Covariates** |  | **Basic model** |  | **Complete model** |  |  |
| --- | --- | --- | --- | --- | --- | --- |
|  | **DD** | **MS** | **DS** | **MS** | **DS** | **Pick** |
| **IRC** | Ref | 20529.9359 | 5834.5368 | 29378.5006 | 13469.2843 |  |
| **Gender** | Ref | 20502.0656 | 5828.4057 | 29586.1042 | 13549.6949 |  |
| **Age** | Ref | 20535.7156 | 5910.8128 | 29177.5699 | 13008.8176 |  |
| **Occlusion site** | Ref | 19770.5688 | 5566.1982 | 29456.7144 | 12100.8912 | Yes |
| **Infarction site** | Ref | 21292.6369 | 6403.6187 | 28879.0626 | 13092.9525 |  |
| **Hypertension** | Ref | 20704.8643 | 6200.1378 | 29095.9377 | 12954.1057 |  |
| **Diabetes mellitus** | Ref | 20908.9929 | 5977.4878 | 28767.9051 | 13191.3380 |  |
| [**Atrial**](javascript:;) [**fibrillation**](javascript:;) | Ref | 20524.9850 | 5834.6834 | 29659.3463 | 13585.7207 |  |
| [**Coronary**](javascript:;) [**heart**](javascript:;) [**disease**](javascript:;) | Ref | 20514.8787 | 6123.8624 | 29507.1517 | 13027.0247 |  |
| [**Hyperlipemia**](javascript:;) | Ref | 21093.2283 | 5869.6092 | 28384.8178 | 13339.7054 |  |
| **Gout** | Ref | 20112.0787 | 5788.7240 | 29781.3932 | 13557.2337 |  |
| **Previous stroke** | Ref | 20242.8311 | 5655.3506 | 29513.6035 | 13555.8402 |  |
| **Smoking** | Ref | 20717.6386 | 5894.1731 | 29759.6161 | 13558.8855 |  |
| **Drinking** | Ref | 20898.2628 | 5961.1101 | 29252.1224 | 13352.5232 |  |
| **Systolic BP** | Ref | 20491.2081 | 5794.5212 | 29102.9461 | 13393.5623 |  |
| **ASPECTS** | Ref | 19074.0110 | 4645.4728 | 28629.2794 * | 12905.4060 * | Yes |
| **Diastolic BP** | Ref | 20390.3060 | 5776.5322 | 29497.9514 | 13510.0260 |  |
| **Pulse** | Ref | 20723.9408 | 5984.4725 | 29326.0320 | 13443.2967 |  |
| **TOAST** | Ref | 20503.6170 | 5849.1773 | 29357.9130 | 13469.6237 |  |
| **IV** | Ref | 22557.2955 | 8045.0559 | 27541.5644 * | 11065.4384 * | Yes |
| **NIHSS** | Ref | 19953.2655 | 4833.9566 | 29386.4271 * | 13659.1059 * | Yes |
| **Threapy** | Ref | 17005.2498 * | 1273.6011 * | 32742.7825 * | 20328.9387 * | Yes |
| **ORT** | Ref | 20705.1122 | 6004.0846 | 28617.1363 | 12729.1692 |  |
| **PRT** | Ref | 28148.1052 * | 14090.0642 * | 24894.3796 * | 7297.0631 * | Yes |
| **DRT** | Ref | 23969.9549 * | 8874.8563 * | 28897.7746 * | 13113.4721 * | Yes |
| [**Recanalization**](javascript:;) | Ref | 20902.3174 | 5622.4643 | 28986.1959 | 13919.7538 |  |

DD: Drive the Doctor; MS: Mothership; DS: Drip and Ship; Ref: Reference; IRC: Initial regression coefficient; BP: Blood pressure; ASPECTS: Albert Stroke Project Early CT Score; TOAST: Trial of Org 10172 in Acute Stroke Treatment; IV: [Intravenous](javascript:;) [thrombolysis](javascript:;); NIHSS: National Institutes of Health Stroke Scale; ORT: onset-to-recanalization time; PRT: puncture-to-recanalization time; DRT: door-to-recanalization time.

* indicates a change of more than 10% compared with the initial regression coefficient.

**Table S18 Baseline data of group A and B**

|  | **group A**  **N=266** | **group B**  **N=83** | ***P*-value** |
| --- | --- | --- | --- |
| **Male(n,%)** | **156 (58.65%)** | **52 (62.65%)** | **0.516** |
| **Age, ‾X(SD)** | **65.29 (13.70)** | **66.77 (12.69)** | **0.382** |
| **NIHSS, M(IQR)** | **16.00 (12.00-20.00)** | **15.00 (11.00-19.00)** | **0.133** |
| **ASPECTS, M(IQR)** | **7.00 (6.00-7.00)** | **7.00 (7.00-8.00)** | **<0.001** |
| **Pulse, ‾X(SD)** | **83.08 (18.59)** | **78.04 (17.28)** | **0.029** |
| **Systolic BP, ‾X(SD)** | **148.70 (23.62)** | **150.17 (21.28)** | **0.613** |
| **Diastolic BP, ‾X(SD)** | **86.49 (16.54)** | **87.77 (15.09)** | **0.531** |
| **Risk factor (n,%)** |  |  |  |
| **Hypertension** | **184 (69.17%)** | **51 (61.45%)** | **0.190** |
| **Diabetes mellitus** | **47 (17.67%)** | **20 (24.10%)** | **0.194** |
| [**Atrial**](javascript:;) [**fibrillation**](javascript:;) | **117 (43.98%)** | **35 (42.17%)** | **0.771** |
| [**Coronary**](javascript:;) [**heart**](javascript:;) [**disease**](javascript:;) | **25 (9.40%)** | **6 (7.23%)** | **0.544** |
| [**Hyperlipemia**](javascript:;) | **84 (31.58%)** | **30 (36.14%)** | **0.439** |
| **Gout** | **9 (3.38%)** | **4 (4.82%)** | **0.546** |
| **Previous stroke** | **49 (18.42%)** | **20 (24.10%)** | **0.257** |
| **Smoking** | **78 (29.32%)** | **22 (26.51%)** | **0.620** |
| **Drinking** | **61 (22.93%)** | **16 (19.28%)** | **0.483** |
| **TOAST(n,%)** |  |  | **0.598** |
| **Large-artery atherosclerosis** | **115 (43.23%)** | **41 (49.40%)** |  |
| **Cardioembolism** | **135 (50.75%)** | **37 (44.58%)** |  |
| **Other/undetermined etiology** | **16 (6.02%)** | **5 (6.02%)** |  |
| **Procedural characteristics** |  |  |  |
| **Occlusion site( (n,%)** |  |  | **0.377** |
| **Intracranial ICA** | **66 (24.81%)** | **27 (32.53%)** |  |
| **MCA** | **166 (62.41%)** | **46 (55.42%)** |  |
| **Vertebral/basilar artery** | **34 (12.78%)** | **10 (12.05%)** |  |
| **Infarction site (n,%)** |  |  | **0.202** |
| **Left** | **139 (52.26%)** | **50 (60.24%)** |  |
| **Right** | **127 (47.74%)** | **33 (39.76%)** |  |
| **IV (n,%)** | **90 (33.83%)** | **49 (59.04%)** | **<0.001** |
| **Therapy(n,%)** |  |  | **0.011** |
| **Stent** | **127 (47.74%)** | **35 (42.17%)** |  |
| **Aspiration** | **31 (11.65%)** | **3 (3.61%)** |  |
| **Stent combined aspiration** | **38 (14.29%)** | **9 (10.84%)** |  |
| **Other** | **70 (26.32%)** | **36 (43.37%)** |  |
| **time indicators, M(IQR)** |  |  |  |
| **ODT** | **170.00 (112.82-269.50)** | **90.00 (56.00-144.00)** | **<0.001** |
| **OPT** | **270.00 (212.50-364.50)** | **230.00 (172.50-289.00)** | **<0.001** |
| **ORT** | **335.00 (269.25-428.25)** | **305.00 (251.50-381.00)** | **0.100** |
| **PRT** | **52.00 (37.00-77.75)** | **82.00 (60.00-110.00)** | **<0.001** |
| **DPT** | **92.50 (50.25-142.75)** | **110.00 (74.50-173.00)** | **0.001** |
| **DRT** | **148.50 (111.00-202.61)** | **210.00 (159.00-278.50)** | **<0.001** |

Group A: DS+MS; Group B: DD; ‾X: mean; SD: standard deviation; M: median; IQR: [interquartile](javascript:;) [range](javascript:;); NIHSS: National Institutes of Health Stroke Scale; ASPECTS: Albert Stroke Project Early CT Score; BP: Blood pressure; TOAST: Trial of Org 10172 in Acute Stroke Treatment; ICA: [Internal](javascript:;) [carotid](javascript:;) artery; MCA: [Middle](javascript:;) [cerebral](javascript:;) [artery](javascript:;); IV: [Intravenous](javascript:;) [thrombolysis](javascript:;); ODT: onset-to-door time; OPT: onset-to-puncture time; ORT: onset-to-recanalization time; PRT: puncture-to-recanalization time; DPT: door-to-puncture time; DRT: door-to-recanalization time.

**Table S19 Outcomes after endovascular thrombectomy of group A and B**

| **Outcomes** | **Group A**  **N=266** | **Group B**  **N=83** | ***P*-value** |
| --- | --- | --- | --- |
| [**Recanalization**](javascript:;) **(n,%)** | **217 (81.58%)** | **68 (81.93%)** | **0.943** |
| **Cost, M(IQR)** | **72947.50 (52633.25-99029.50)** | **64258.00 (45832.00-88586.50)** | **0.076** |
| **sICH(n,%)** | **51 (19.17%)** | **19 (22.89%)** | **0.460** |
| **asICH (n,%)** | **64 (24.06%)** | **19 (22.89%)** | **0.827** |
| **mRS, M(IQR)** | **4.00 (2.00-6.00)** | **4.00 (2.00-5.00)** | **0.605** |
| **mRS(n,%)** |  |  | **0.030** |
| **0** | **17 (6.39%)** | **3 (3.61%)** |  |
| **1** | **48 (18.05%)** | **10 (12.05%)** |  |
| **2** | **22 (8.27%)** | **14 (16.87%)** |  |
| **3** | **35 (13.16%)** | **9 (10.84%)** |  |
| **4** | **54 (20.30%)** | **15 (18.07%)** |  |
| **5** | **15 (5.64%)** | **12 (14.46%)** |  |
| **6** | **75 (28.20%)** | **20 (24.10%)** |  |
| **Mortality(n,%)** | **75 (28.20%)** | **20 (24.10%)** | **0.464** |
| **Poor outcome (n,%)** | **179 (67.29%)** | **56 (67.47%)** | **0.976** |

Group A: DS+MS; Group B: DD; sICH: Symptomatic intracranial hemorrhage transformation; asICH: Asymptomatic intracranial hemorrhage transformation; mRS: modified Rankin Scale.

**Table S20 Multiple regression analysis of group A and B**

|  | **Unadjusted**  ***OR*/*β*(95%CI) *P*** | **Model I**  ***OR*/*β*(95%CI) *P*** | **Model II**  ***OR*/*β*(95%CI) *P*** |
| --- | --- | --- | --- |
| **sICH** |  |  |  |
| **group A** | **1.0** | **1.0** | **1.0** |
| **group B** | **1.25 (0.69, 2.27) 0.4607** | **1.24 (0.68, 2.25) 0.4862** | **2.08 (0.88, 4.91) 0.0955** |
| **Poor outcome** |  |  |  |
| **group A** | **1.0** | **1.0** | **1.0** |
| **group B** | **1.01 (0.60, 1.71) 0.9761** | **0.97 (0.56, 1.65) 0.8975** | **1.79 (0.84, 3.84) 0.1340** |
| **Mortality** |  |  |  |
| **group A** | **1.0** | **1.0** | **1.0** |
| **group B** | **0.81 (0.46, 1.43) 0.4644** | **0.76 (0.43, 1.37) 0.3657** | **1.61 (0.64, 4.08) 0.3130** |
| **Cost** |  |  |  |
| **group A** | **0** | **0** | **0** |
| **group B** | **-10530.44 (-24703.60, 3642.73) 0.1462** | **-10594.50 (-24842.80, 3653.79) 0.1459** | **-19438.86 (-35977.79, -2899.94) 0.0219** |

Group A: DS+MS; Group B: DD; Model I: adjusted for age and gender; Model II: adjusted the variables with statistical differences in univariate analysis Table S5-8 (*P*<0.1) and the covariates selected in Table S14-17 in the appendix; sICH: Symptomatic intracranial hemorrhage transformation.
